# STING signaling in vestibular macrophages underlies Ménière’s disease pathogenesis

**DOI:** 10.1186/s12974-026-03812-4

**Published:** 2026-04-14

**Authors:** Jiahui Liu, Na Li, Yurong Mu, Yongdong Song, Yao Lu, Xiaoxuan Xu, Na Zhang, Jing Wang, Xiaofei Li, Hanyue Wang, Yan Wang, Zhaomin Fan, Yafeng Lyu, Yuechen Han, Daogong Zhang, Haibo Wang

**Affiliations:** 1 Department of Otolaryngology-Head and Neck Surgery, Shandong Provincial ENT Hospital, Shandong University, Jinan, Shandong 250022 China; 2 Shandong Second Provincial General Hospital & Shandong Institute of Otorhinolaryngology & Shandong Key Laboratory of Deafness and Vertigo &, Shandong Clinical Medical Research Center for Otolaryngological Diseases & Shandong Key Laboratory of Vertigo and Vestibular Medicine, Jinan Key Laboratory of Vertigo and Balance Medicine, Jinan, Shandong 250022 China; 3 Center of Clinical Laboratory, Shandong Second Provincial General Hospital, Jinan, Shandong 250022 China

**Keywords:** Ménière’s disease, STING, CUL4B-DDB1-ROC1 complex, Hearing loss, Vertigo

## Abstract

**Background:**

Ménière’s disease (MD) is a disabling inner-ear disorder. Although the key pathologic alteration of MD is characterized as endolymphatic hydrops (EH), the underlying pathogenic mechanism remains unclear, and no definitive curative treatments are available. Emerging evidence implicates oxidative stress and innate immunity as key contributors to MD pathogenesis.

**Methods:**

Human vestibular end organs were collected to characterize the reactive oxygen species (ROS) levels and the STING signaling activation, findings that were further corroborated in an LPS-induced EH mouse model. Transfection and transwell co-culture were performed to investigate the feedback loop between sensory epithelial cells and macrophages in vitro. Molecular biology experiments were performed to elucidate the underlying regulatory mechanisms. Additionally, STING and CUL4B deficiency mice were used to explore potential therapeutic targets in vivo.

**Results:**

The oxidative stress, cytoplasmic dsDNA leakage, and STING activation were elevated in both MD patients and LPS-induced EH mouse model. The dsDNA released from ROS-damaged hair cells activates STING in vestibular macrophages. STING-specific knockout in macrophages alleviates EH, audio-vestibular dysfunction, and interferon-stimulated genes expression in mouse model. The CUL4B-DDB1-ROC1 complex ubiquitinates STING at K370, promoting its degradation and inhibiting activation. Notably, CUL4B deficiency exacerbates LPS-induced EH, audio-vestibular dysfunction, and interferon-stimulated genes (ISG) upregulation, while double conditional knockout reverses these effects.

**Conclusion:**

Our study demonstrates that STING activation in vestibular macrophages contributes to audio-vestibular dysfunction and modulating this pathway has promising beneficial effects on audio-vestibular function in EH mice model, which is a potential therapeutic strategy for MD.

**Graphical Abstract:**

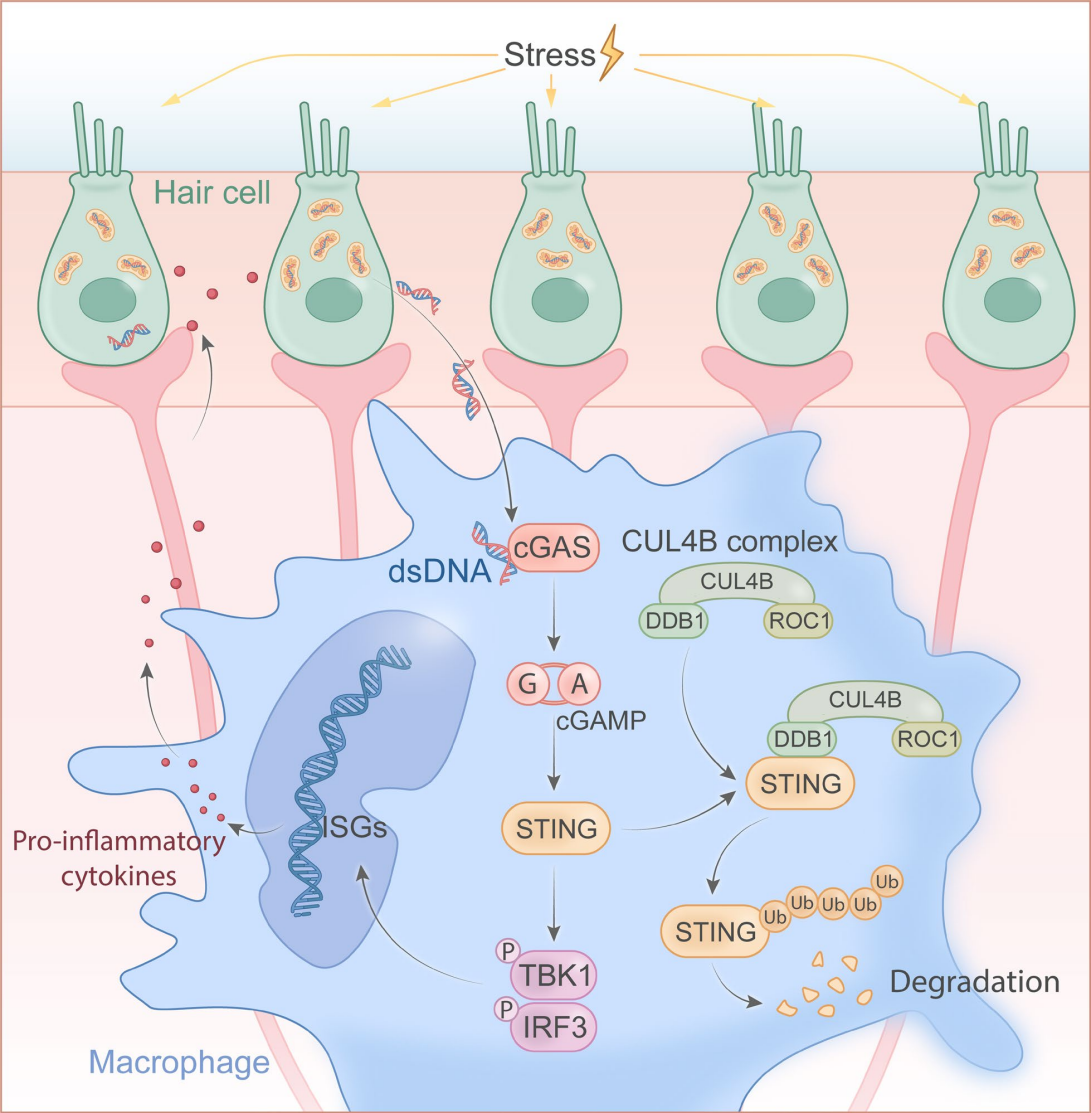

**Supplementary Information:**

The online version contains supplementary material available at 10.1186/s12974-026-03812-4.

## Introduction

Ménière’s disease (MD) is a chronic inner-ear disorder characterized by recurrent vertigo, fluctuating sensorineural hearing loss, tinnitus, and aural fullness, imposing a substantial personal and socioeconomic burden [1]. The most distinctive pathological feature of MD is endolymphatic hydrops (EH), which reflects abnormal fluid accumulation and pressure within the membranous labyrinth, stimulating and potentially damaging the sensory epithelium [2, 3]. Despite decades of investigation, MD lacks a definitive cure, and current therapies remain largely symptomatic [4]. Multiple factors—including immune dysregulation, oxidative stress, metabolic disturbances, and anatomical or genetic predisposition—have all been implicated, but the precise pathogenic cascade remains elusive [5, 6].

Recent advances in genetic and immunological research have increasingly positioned dysregulated immunity, particularly innate immune activation, as a central driver of MD pathogenesis [5, 7]. Genetic studies have identified variants in immune-related genes like *TNF-α*, [8] *NF-KB1 *[9], and *MIF *[10], which contribute to the activation of the inflammasome and the release of pro-inflammatory cytokines. Moreover, recent multi-omics analyses have delineated three distinct immunological profiles in MD: a monocyte-driven autoinflammatory profile characterized by elevated IL-1β, an allergic profile associated with high IgE and type 2 cytokines, and a subgroup with normal cytokine levels [11, 12]. Beyond systemic inflammation, studies have highlighted the resident macrophages, the predominant immune cells in the inner ear, as critical effectors of the inflammatory cascade, with NLRP3 inflammasome activation driving the release of IL-1β [13]. Although these findings point to a pivotal role of innate immunity in MD, the precise biological mechanisms and the triggers of this immune response remain poorly understood.

Excessive reactive oxygen species (ROS) in sensory epithelial cells can damage mitochondria, causing the release of double-stranded DNA (dsDNA) and other damage-associated molecular patterns (DAMPs) into the cytosol [14]. These endogenous DNA fragments serve as innate immune stimuli and are sensed by cyclic GMP-AMP synthase (cGAS), a key cytosolic sensor of dsDNA [15]. Upon binding DNA, cGAS undergoes phase separation and synthesizes 2′3′-cyclic GMP-AMP (cGAMP), a second messenger that activates the ER-resident adaptor STING.^16^ Activated STING triggers downstream signaling through the TBK1–IRF3–NF-κB axis, leading to the transcription of type I interferons (IFN-Is), interferon-stimulated genes (ISG), and proinflammatory cytokines such as *TNF-α*, *IL-6*, and *IL-1β *[17]*.* Thus, the cGAS-STING pathway converts oxidative-stress-induced self-DNA fragmentation into a potent innate immune response [18]. Beyond its classical role in host defense, STING signaling has been implicated in a wide range of physiological and pathological processes, such as autoimmunity [19], cellular senescence [20], autophagy [21], inflammation [22], and cancer immunity [23]. Notably, patients with MD exhibit evidence of systemic oxidative stress—for example, lymphocytes from MD patients show elevated protein carbonyls and 4-hydroxynonenal (4-HNE) levels [24, 25], and the oxidative stress marker iNOS is highly expressed in hydropic vestibular organs [26]. Furthermore, genome-wide CRISPR-Cas9 screen uncovered mitochondrial oxidative phosphorylation dysfunction-related gene *FAM136A* associated with MD [27, 28]. However, the precise role of oxidative stress and STING signaling in MD progression is poorly understood, and whether targeting STING prevents or delays MD progression remains to be elucidated.

In the present study, we demonstrate that dsDNA released from ROS-damaged hair cells robustly activates STING in inner-ear macrophages, that the CUL4B-DDB1-ROC1 E3-ligase (CRL4B) complex serves as an intrinsic brake by targeting STING for degradation, and that genetic STING inhibition preserves audio-vestibular function and mitigates EH in LPS-induced mice model. Taken together, our data position cGAS-STING signaling as the key point between sensory-epithelial damage and macrophage-driven inflammation, and nominate STING as a tractable therapeutic target for MD.

## Materials and methods

### Patients and tissue samples

Fifteen MD patients aged 36–87 years (average age 64·1 years) at Shandong Provincial ENT Hospital affiliated to Shandong University from January 2022 to December 2024 were enrolled in this study. These patients had been diagnosed with MD according to the 2015 Diagnostic criteria for Menière’s disease [29], Control group consisted of 12 patients with vestibular schwannoma (VS) aged 43–70 years (average age 58·7 years) with no history of sudden vertigo. The demographic and socioeconomic indicators of the patients and controls were not significantly different (Table S1). The ampullae and utricles were sampled from MD patients who accepted labyrinthectomy and VS patients who accepted tumour resection via the translabyrinthine route. Informed consent was obtained from all participants prior to surgery. Tissues were immediately snap-frozen and transported in liquid nitrogen, or fixed in 4% paraformaldehyde (PFA) for subsequent processing.

### Animal studies

Wild-type C57BL/6 mice (male, 6–8 weeks old) were purchased from Jinan Pengyue experimental animal breeding Co., Ltd. *Sting*^*fl/fl*^ mice were purchased from GemPharmatech Co., Ltd., and *Cul4b*^*fl/Y*^ mice were provided by Dr. Yaoqin Gong from Shandong University. To specifically delete genes in the myeloid cell lineage, we used *Lyz2*-cre mice to generate *Sting* and/or *Cul4b* conditional knockout mice *(Sting*^*cKO*^ mice, *Cul4b*^*cKO*^ mice, *and Sting*^*cKO*^*Cul4b*^*cKO*^ mice). Mice were housed at 20–22°C with a humidity of 40–70%, under a 12 h–12 h light-dark cycle, and had unlimited access to food and water. Male mice aged 6–8 weeks were used in the experiments. Littermates were randomly allocated to experimental groups, and all experiments were performed single-blindly at the respective ages specified.

To establish EH model, mice were subjected to modeling by postauricular injection of lipopolysaccharide (LPS, 2 mg/kg, Sigma) once a day for 3 consecutive days as previously described [13]. Mice in the control group were injected with an equal amount of 0·9% saline. The hearing and vestibular functions of the mice were evaluated on the 5th day after the initial injection.

### Ethics

The Ethics Committee of Shandong Provincial ENT Hospital affiliated to Shandong University approved this study (IRB-XYK-20220405) and the study was conducted strictly according to the Declaration of Helsinki as revised in 2013.

All animal procedures carried out in this study were in strict compliance with the regulations of the Animal Care Committee of Shandong University (Jinan, China) (No. ECAESDUSM 20,123,011) and conformed to the Guidelines for the Care and Use of Laboratory Animals issued by the National Institutes of Health.

### Cell culture and transfection

Primary vestibular resident macrophage-like (VRML) cells from the vestibular end organs (VEOs) of postnatal day 3 (P3) C57BL/6 mice were collected as described previously [30]. Briefly, freshly isolated VEOs were rapidly cut into small pieces, which were cultured in melanocyte medium (MelM, Sciencell, 2201) with 1% melanocyte growth supplement and penicillin/streptomycin (Gibco, 15140122, 10,000 units/mL ) in a 37°C incubator with 5% CO_2_ for 7 days. The VRML cells were expanded, further selected, and validated based on CD68 and IBA1 expression (Fig. S1A).

HEI-OC1 cells were cultured in a 33°C incubator with 10% CO_2_ in Dulbecco’s Modified Eagle’s Medium (DMEM, Gibco, 12491015) supplemented with 10% fetal bovine serum (FBS, Gibco, A5669701). HEK-293T cells were cultured in a 37 °C incubator with 5% CO_2_ in DMEM supplemented with 10% FBS and penicillin/streptomycin. THP-1 cells (Procell, CL-0233) were cultured in a 37 °C incubator with 5% CO_2_ in Roswell Park Memorial Institute (RPMI)-1640 (Gibco, 11875119) medium supplemented with 10% FBS, and penicillin/streptomycin. Lipofectamine^®^ RNAiMAX Reagent (Invitrogen, 13778-150) or PEIpro (Polyplus, 115 − 0015) was used for the transfection of siRNA or plasmids according to the manufacturer’s instructions. For stimulation, cells were treated with H_2_O_2_ (mi88597, 500 µM) for 24 h, EdU (5-ethynyl-2’-deoxyuridine, beyotime, C0071S, 10 µM) for 2 h, cGAMP (Invitrogen, tlrl-nacga23, 2 µg/mL) for 2 h, MG132 (MCE, HY-13259, 25 µM) for 4 h, or other indicated in the main text. Saline or other appropriate vehicle controls were used as corresponding controls for each stimulant.

### DHE staining

For human tissue, snap-frozen ampullae and utricles were transported in liquid nitrogen to the laboratory, cryopreserved, and then subjected to dihydroethidium (DHE) staining. For P3 mice, the basilar membranes, ampullae and utricles were dissected out under the microscope, attached to a round coverslip using Cell-Tak adhesive (Corning, 354240), cultured in DMEM/F12 containing 10% FBS and 100 µg/mL ampicillin (Solarbio, A1170), stimulated with LPS (200 ng/mL ) for 3 h and ATP (Gibco, 11320033) for 30 min. Tissues were incubated with 10 µM DHE fluorescent probe (Thermo Fisher Scientific, D1168) at 37°C in the dark for 30 min. Then, samples were fixed with 4% PFA and subsequently visualized under a laser scanning confocal microscope (Leica SP8; Leica, Germany). Laser power, exposure and detector gain were strictly held constant within each independent experiment to ensure the validity of cross-sample imaging comparisons. All analyses were conducted by a blinded observer. Images were converted to 8-bit grayscale and analyzed using ImageJ version 1·54k. Equal-sized regions of interest (ROIs) were manually drawn based on structures of basilar membranes, ampullae and utricles. 3–5 non-overlapping ROIs were randomly selected per sample.

### Immunofluorescence staining (IF)

The paraffin sections were baked, dewaxed, and subjected to antigen retrieval. After that, they were permeabilized in 0·5% Triton X-100 for 30 min and then immunoblocked with 5% donkey serum for 1 h. The frozen sections were permeabilized and immunoblocked. For adult mice, the inner ear was isolated, fixed with 4% PFA overnight, and decalcified in 10% ethylenediaminetetraacetic acid (EDTA). The basilar membranes, ampullae, and utricles were dissected and mounted on circular coverslips using Cell-Tak adhesive. For cells, approximately 10^5^ cells were plated on a round coverslip. Treated cells were fixed, permeabilized, and immunoblocked. The specimens were incubated with different primary antibodies at 4 °C overnight. The primary antibodies were anti-dsDNA (Abcam, Cat# ab27156, RRID: AB_470907, used 1:400), anti-TOMM20 (Abcam, Cat# ab78547, RRID: AB_2043078, used 1:400), anti-STING (Thermo Fisher Scientific, PA5-20782, used 1:400), anti-IBA1 (Abcam, Cat# ab5076, RRID: AB_2224402, used 1:200), anti-CD68 (Proteintech, Cat# 28058-1-AP, RRID: AB_2881049, used 1:200), anti-MYO7A (Santa Cruz Biotechnology Cat# sc-74516, RRID: AB_2148626, used 1:50), anti-CtBP2 (BD, Cat# 612044, RRID: AB_399431, used 1:200), anti-CUL4B (Sigma-Aldrich, Cat# C9995, used 1:200), and anti-DDB1 (Proteintech, Cat# 11380-1-AP, RRID: AB_2088808, used 1:200). The next day, cells were incubated with fluorescent secondary antibodies (Invitrogen, 1:1000) in the dark at room temperature for 1 h. Finally, the samples were mounted on the slide with Prolong Gold with DAPI (Invitrogen, P36931) and observed under a laser scanning confocal microscope. Laser power, exposure and detector gain were strictly held constant within each independent experiment to ensure the validity of cross-sample imaging comparisons. Minimal post-acquisition processing, including background subtraction and non-saturating contrast adjustment, was applied uniformly across all groups solely to enhance signal clarity without altering original experimental data Images were converted to 8-bit grayscale and analyzed using ImageJ version 1·54k. 3–5 non-overlapping ROIs were randomly selected based on structures or as indicated in the relevant figure legend. All analyses were conducted by a blinded observer.

### TUNEL staining

Basilar membranes of all cochlear turns (apical, middle, and basal), as well as ampullae, and utricles, were dissected from mice and mounted. TUNEL staining (Invitrogen, C10617) was performed according to the manufacturer’s instructions. Subsequently, samples were co-stained with DAPI and MYO7A antibodies and visualized under a laser scanning confocal microscope. 3–5 non-overlapping 150 μm × 150 μm square ROI were acquired per sample. All analyses were conducted by a blinded observer.

### Electron microscopy

For transmission electron microscopy, utricles obtained from MD and VS patients were fixed in 3% glutaraldehyde fixative solution, then fixed in 1% osmic acid (OsO_4_), dehydrated, soaked, and embedded in Epon 812, ultrathin radial sections were stained with lead citrate and uranyl acetate and observed using a transmission electron microscope (JEOL-1200EX, JEOL, Japan).

For immunoelectron microscopy (IEM), tissues were fixed in an IEM-specific fixative and 1% OsO_4_, dehydrated, embedded in resin and sectioned at ultrathin thickness. Sections were blocked with 1% bovine serum albumin (BSA) and incubated with anti-dsDNA antibody overnight at 4°C. After incubation with 10 nm colloidal gold secondary antibody (Invitrogen, A-31561), sections were observed under a transmission electron microscope.

For scanning electron microscopy analysis, the inner ears were removed from animals and the cochleae dissected, immersed in 3% glutaraldehyde fixative solution, and decalcified, the basilar membranes were finely dissected, post-fixed in 1% OsO_4_ and then dehydrated, critical point dried, sputter coated with gold, and imaged on a scanning electron microscope.

### Quantitative real-time PCR (qRT-PCR)

The total RNA from mouse inner ear and cell lines was extracted using Trizol reagent (Invitrogen) according to manufacturer’s protocols. 1 µg of RNA was reverse-transcribed into cDNA using a high-capacity DNA reverse transcription kit following the manufacturer’s instructions. Quantitative RT-PCR was performed using SYBR Premix Ex Taq (TaKaRa Bio, RR420A) with *Gapdh* as the housekeeping gene. All data were analyzed using an Eppendorf Realplex 2. PCR primers for the genes are listed in Table S2.

### Western blot

Total protein was extracted with RIPA lysis buffer containing a proteinase inhibitor cocktail from cells, ground tissue and mouse inner ear, and denatured at 98°C for 10 min after concentration determination. Protein samples were separated on 10% SDS-PAGE gel and transferred to polyvinylidene difluoride (PVDF) membranes. The membranes containing protein samples were blocked with 5% skim milk at room temperature for 1 h, then incubated with primary and corresponding secondary antibodies. The primary antibodies were anti-TBK1 (Abcam, Cat# ab40676, RRID: AB_776632, used 1:1000), anti-p-TBK1 (Cell Signaling Technology, Cat# 5483, RRID: AB_10693472, used 1:1000), anti-IRF3 (Abcam, Cat# ab68481, RRID: AB_11155653, used 1:1000), anti-p-IRF3 (Cell Signaling Technology Cat# 29047, RRID: AB_2773013, used 1:1000), anti-STING (Cell Signaling Technology, Cat# 13647, RRID: AB_2732796, used 1:2000), anti-p-STING (Cell Signaling Technology Cat# 50907, RRID: AB_2827656, used 1:1000), anti-CUL4B (Sigma-Aldrich Cat# C9995, RRID: AB_1840781, used 1:2000), anti-DDB1 (Proteintech, Cat# 11380-1-AP, RRID: AB_2088808, used 1:2000), anti-ROC1 (Proteintech, Cat# 14895-1-AP, RRID: AB_2179719, used 1:2000), anti-Ub (Cell Signaling Technology Cat# 3936, RRID: AB_331292, used 1:1000), anti-K48-Ub (Cell Signaling Technology Cat# 12805, RRID: AB_2798031, used 1:1000), anti-K63-Ub (Cell Signaling Technology Cat# 5621, RRID: AB_10827985, used 1:1000), and anti-ACTIN (Thermo Fisher Scientific, Cat# MA5-11869, RRID: AB_11004139, 1:1,000). Finally, the protein signals were detected using an SuperSignal™ West Pico PLUS (Thermo Fisher Scientific, 34579) and analyzed using ImageJ software.

### Flow cytometry

Cell apoptosis was assessed using an Annexin V-FITC Apoptosis Detection Kit (BD Biosciences, 556547). The cells were collected, washed with PBS, and gently resuspended in 195 µL of binding buffer. Subsequently, 5 µl of Annexin V-FITC and 10 µl of propidium iodide (PI) were added and incubated for 15 min at room temperature in the dark. 10,000 cells from each group were analyzed using an LSRII flow cytometer with BD FACSDiva software version 9 (BD Biosciences, USA), and the data were further analyzed using FlowJo software (FlowJo, LLC).

### Cytosolic dsDNA isolation and transfection

HEI-OC1 cells were treated with 500 µM H_2_O_2_ for 24 h to induce DNA damage. To avoid the contamination of mitochondrial DNA released into the cytosol, mitochondrial DNA and cytosolic DNA were isolated from HEI-OC1 cells by using a Mitochondria Isolation Kit (Thermo Fisher, 89801) according to the manufacturer’s instructions. The supernatant, which contained cytosolic DNA, was transferred to a new tube, and the pellet containing mitochondria was discarded. We then used the Monarch^®^ PCR & DNA Cleanup Kit (New England Biolabs, T1030S) to remove any remaining mitochondria or lysosome contamination. The concentration of cytosolic dsDNA was measured by using a PicoGreen Assay Kit (Thermo Fisher, P7589) according to the manufacturer’s instructions. Then, we transfected VRML cells with the dsDNA (2 µg/mL) for 24 h, isolated from HEI-OC1 cells by using PEIpro as a carrier.

### Detection of EdU phagocytosis by VRML cells in transwell co-culture system

HEI-OC1 cells were first labeled with EdU for 2 h. These EdU-labeled HEI-OC1 cells were then added into the insert of a 24-well transwell (0·4 μm, Thermo Fisher Scientific, 141002) and treated with 500 µM H_2_O_2_ for 24 h to induce cell injury. VRML cells were pre-plated onto the 24-well plate. The VRML cells and HEI-OC1 cells were co-cultured without direct cell contact. A control group consisted of HEI-OC1 cells that were identically processed but not exposed to H₂O₂.

### EdU-labeled cytosolic dsDNA posterior semicircular canal (PSCC) microinjection

EdU-labeled cytosolic dsDNA was isolated and injected into the inner ear of adult mice through PSCC. After anesthetizing the mice, the postauricular region was carefully shaved, and a 1 cm incision was made behind the ear to expose PSCC by separating the pinna and sternocleidomastoid muscle. A small hole was made in the PSCC using a dental drill. The hole was then connected to a Nanoliter 2000 micromanipulator (WPI, Micro2T, USA) via a glass micropipette (WPI, 504949, USA) that was connected to a fine polyimide tube. The tube tip was inserted into the PSCC, and the solution was injected at a controlled rate of 50 nL/s, with a total volume of 1·5 µL administered. Following injection, the skin was carefully aligned and the wound bonded with tissue adhesive (3 M Vetbond, 1469SB). Animals undergoing sham operation were used as controls.

### Quantitative assessment of changes in endolymphatic space in cochlea

Frozen sections of the mouse inner ear were stained with hematoxylin and eosin (H&E), dehydrated through an ethanol gradient, and mounted with neutral gum. The cochlear turns from the base to the apex were designated as half-turns I to IV in sequence. Both the length of the stretched Reissner’s membrane (L) and the ideal length of the Reissner’s membrane (L*) were measured in each cochlea, and the increase ratio of the length change of the Reissner’s membrane (IR-L = L/L* x 100%) was calculated to evaluate the EH levels, as previously described [31]. The thickness of stria vascularis (SV) and number of fibrocytes within the spiral ligament (SLi) in each half-turns was measured in ROIs that were delineated by a manual tracing of the structures. These values were analyzed using ImageJ software.

### Auditory brainstem evaluation (ABR)

The mice were anesthetized by intraperitoneal injection of xylazine (10 mg/kg) and ketamine (100 mg/kg). Inside the soundproof barrier, a TDT System 3 (Tucker-Davis Technologies) was used to record the responses with click sound stimuli and tone burst stimuli at frequencies of 4, 8, 12, 16, 24, and 32 kHz. Each record had 512 repetitions of the stimuli. Needle electrodes were inserted into the subcutaneous tissue of the top of the mouse’s head (recording electrode), the subcutaneous tissue of the mastoid region behind the ipsilateral ear (reference electrode), and the subcutaneous tissue behind the contralateral ear (ground electrode). The sound intensity started at 90 dB sound pressure level (SPL), and then was decreased successively in gradients of 10 dB until no waveform was observed. Then, the sound intensity increased by 5 dB. The ABR threshold was determined at each frequency. This threshold referred to the minimum sound pressure level at which one or more distinguishable waveforms could be clearly identified by visual inspection, thus enabling a reliable ABR record to be obtained. This process was repeated at low sound pressure levels near the threshold to ensure the consistency of the waveforms. The ABR test was performed on a mouse by the same individual both before and after the model establishment, to obtain the mouse’s ABR threshold shift.

### Distortion product otoacoustic emissions (DPOAE)

Otoscopy was used to examine the external auditory canal and tympanic membrane, excluding tympanic membrane and middle ear abnormalities. The anesthetized mice were placed in a soundproof chamber, with a probe sealed in the external auditory canal. Testing began after stimulation signals stabilized, with ƒ1 and ƒ2​ tones were delivered by a probe with two independent intra-canal speakers. The TDT System 3 was employed to measure 2ƒ1-ƒ2 (with ƒ2/ƒ1 = 1·22, and ƒ2 set 5 dB lower than ƒ1) at 12 kHz and 16 kHz frequencies. The DPOAE threshold was defined as the level at which 2ƒ1-ƒ2 exceeded the noise floor by at least 3 dB.

### Endocochlear potential (EP) measurement

For EP measurement, the anesthetized mouse was fixed on a heating pad and placed ventrally in a soundproof chamber. A midline neck incision was made, subcutaneous tissues and muscles were separated to expose the trachea, and a simple tracheotomy was performed. The soft tissues attached to the surface of the acoustic vesicle were dissected. After adequate hemostasis, the acoustic vesicle was opened to fully expose the cochlear basal turn. A small hole was created at the basal turn using a dental drill, with care taken to avoid membranous labyrinth damage. EP was recorded using a pulled-glass capillary microelectrode filled with 150 mM KCl, which was mounted on a micromanipulator and advanced through the hole into the scala media via the stria vascularis while monitoring the potential. The reference (ground) electrode was embedded in the ipsilateral neck soft tissue, signals were amplified and recorded with the patch clamp amplifier (HEKA EPC10 USB).

### Vestibular evoked myogenic potential (VEMPs)

After anesthetizing the mice, click-evoked VEMP recordings were carried out, and electromyogram potentials were recorded simultaneously. The necks of the mice were hyperextended, and the incisors were fixed with a suspension wire. The mice were kept in a prone position with their heads raised and all four limbs fixed. During the recording process, a platinum needle electrode was inserted into the extensor muscles of the mice’s necks, the reference electrode was placed in the cervico-occipital region at the midline, and then the ground electrode was placed subcutaneously on the back. The VEMP test was performed on each mouse at a stimulus intensity of 100 dB normalized hearing level (nHL) and recorded by Neuro-Audio system (Neurosoft). The repeatability of the waveform was verified through consecutive tests (more than 3 times). The latencies and amplitudes of the negative and positive peaks were measured.

### Vestibular ocular reflexes (VOR)

VOR test was conducted according to the method previously described [32]. Briefly, the mice were placed in a non-invasive animal fixation device. The horizontal eye position signals were recorded using a vestibular function testing system (VFT) based on binocular video oculography (VOG), provided by the team of Professor Fangyi Chen (Southern University of Science and Technology). An infrared camera equipped with a zoom lens (MI, China) was installed on the translational platforms at an angle of 45 degrees relative to the anteroposterior axis of the mouse. The lighting for video recordings was achieved by using two near-infrared light-emitting diode lamps (940 nm, Chuntaxin, China) connected to the camera. The eye movement tracker monitored the ROI at a speed of 60 frames per second. The ROI, which contained the pupil, in each frame was automatically selected using a template-matching method. Then, an elliptical fitting was used to determine the center of the pupil and extract the horizontal component related to the eye movements. The obtained translation distances were converted into the angle of eye rotation for calibration purposes. To measure the responses of the vestibular-ocular reflex, horizontal rotations were carried out at a speed of 20 degrees per second (frequencies of 0·2, 0·5, 0·8, 1·0, and 1·6 Hz). The exported data on the eye position were subjected to a Fourier transform using the MATLAB 2016b software to obtain the data on the amplitude of the eye movements. The gain of the vestibular-ocular reflex was calculated as the amplitude ratio between the response and the stimulus.

### Rotarod test

The mice were placed on an electric rotating rod (ZH–600B, Huaibei Zhenghua Biological Instruments Co., Ltd.) with a maximum speed of 30 rpm. Then the speed was gradually increased to 30 rpm over a period of 2 min. Each mouse was trained twice a day for 3 days and tested twice. Subsequently, the average time that the mouse spent on the rotating rod before falling off in the two trials was recorded for further analysis.

### Open field test

The mice were placed in an open field chamber with a length and width of 50 cm and a height of 40 cm, allowing them to move freely. A camera was used to record the behavioral activities of the mice within a 10 min period. The movement trajectories and angular velocities of the mice were analyzed by the VisuTrack software from Shanghai Xinruan Information Technology Co., Ltd. (Shanghai, China). The open field area was cleaned with 75% ethanol between each test.

### Co-immunoprecipitation (Co-IP) assays and IP-MS/MS

For the Co-IP experiment, cells were washed twice with pre-cooled PBS and lysis in buffer (50 mM Tris–HCl; pH 7·4, 150 mM NaCl, 1 mM EDTA, 0·5% NP-40, 0·25% sodium deoxycholate, and protease-inhibitor cocktail) at 4 °C for 30 min. The mixture was centrifuged at 12,000 × g for 15 min and the supernatant was collected for further use. An appropriate amount of the cell lysate was mixed with primary antibody or normal rabbit/mouse immunoglobulin G (IgG) and incubated with rotation at 4 °C overnight. Protein A/G magnetic beads were added to the solution and incubated with rotation at 4 °C for 2 h to enable the capture of immune complexes. After denaturing and washing steps, the complexes were subjected to subsequent SDS-PAGE operations, followed by silver staining (Thermo Fisher, 24612) according to the manufacturer’s instructions. Samples in the gel were excised into multiple gel slices and sent to PTM BIO (Hangzhou, China) for MS/MS protein identification, or sent to Applied Protein Technology Co., Ltd. (Shanghai, China) for LC-MS/MS analysis to identify ubiquitination sites.

### GST pull-down assay

Expression vectors were constructed with a GST tag and the target protein, and their expression was induced in BL21 (Vazyme) *Escherichia coli*. Cells were lysed by ultrasonic disruption and purified the protein using glutathione Sepharose 4B beads (GE Healthcare). In vitro transcription and translation experiments were carried out using rabbit reticulocyte lysate (TNT system, Promega) according to the manufacturer’s recommendations. The beads were incubated with the in vitro translated proteins with rotation at 4 °C overnight, then washed with the binding buffer. The eluates were analyzed by SDS-PAGE.

### Statistical and reproducibility

All statistical analyses were performed using SPSS 13·0 software (SPSS Inc., USA). Data are presented as the mean ± standard error (SEM). Two-tailed unpaired Student’s *t*-test was used for comparisons between two groups. One-way analysis of variance (ANOVA) followed by Tukey’s test was performed in multiple group comparisons. Two-way ANOVA followed by Fisher’s LSD post hoc test was performed for frequency-specific physiological measurements (e.g., ABR thresholds, VOR gains). Fisher’s exact test was used for comparing patient characteristics. Differences with a *P*-value < 0·05 were considered to be statistically significant.

### Role of funders

The National Natural Science Foundation of China, Taishan Scholars Program of Shandong Province, Natural Science Foundation of Shandong Province, Shandong Province medical health science and technology project, and China Postdoctoral Science Foundation provided grants to support the project. No funders had any role in study design, data collection, data analyses, interpretation, or writing of report.

## Results

### Sensory epithelial cell DNA leakage and STING signaling activation in MD patients and LPS-induced EH mice model

**Fig. 1:**
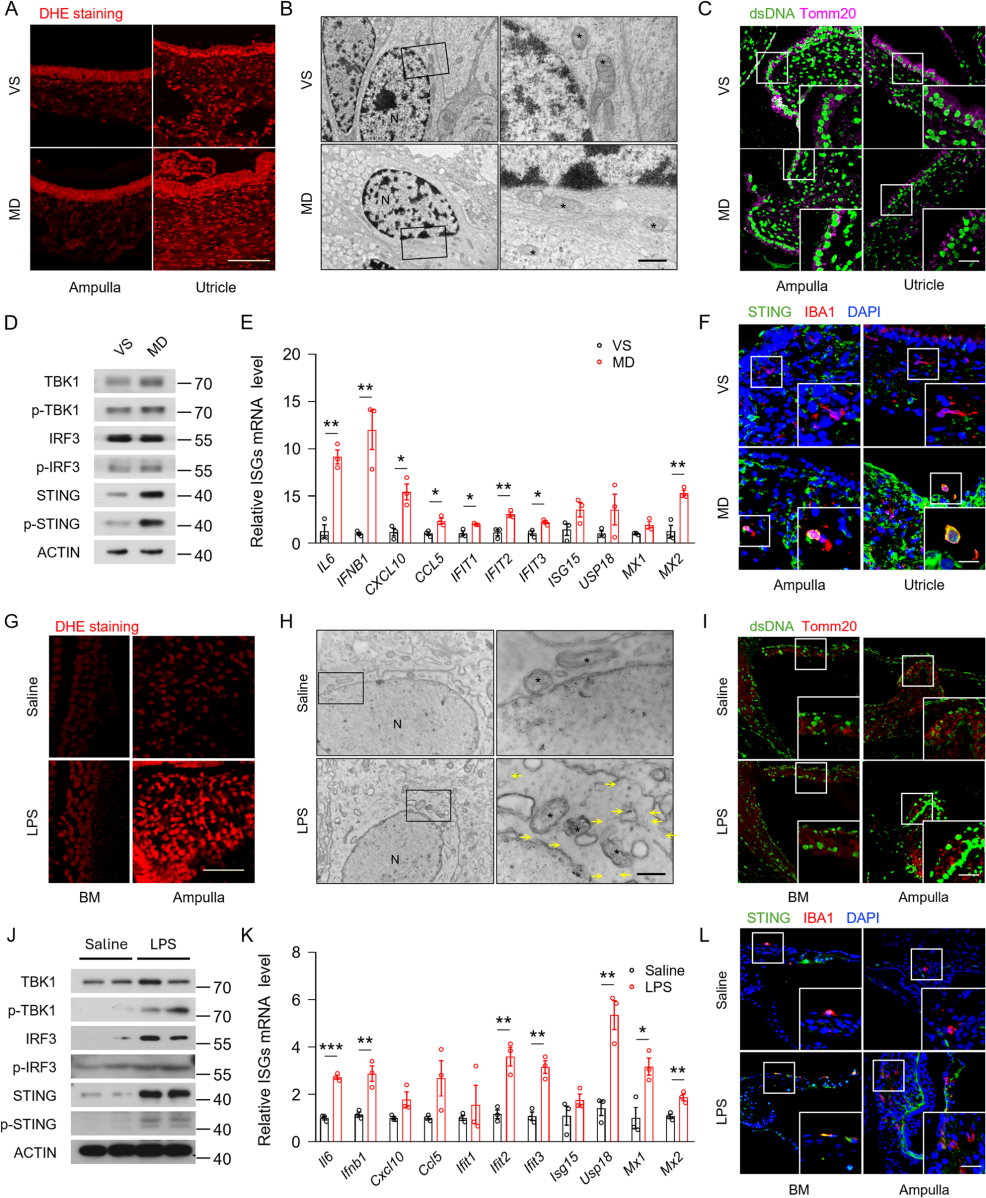
Sensory epithelial cell DNA leakage and STING signaling activation in VEOs of MD patients and inner ear of LPS-induced EH mice model. **A** Images showing DHE staining in the ampullae and utricles of VS and MD patients (*n* = 3 patients per group, scale bar = 100 μm). **B** Transmission electron images showing mitochondrial (*) injury in hair cells of VS and MD patients (*n* = 3 patients per group, scale bar = 500 nm). **C** Representative immunofluorescence images showing dsDNA (green) and Tomm20 (magenta) staining in the ampullae and utricles of VS and MD patients (*n* = 6 patients per group, scale bar = 12·5 μm). **D** Western Blot to detect the expression of key proteins of the cGAS-STING signaling pathway in the ampullae and utricles of VS and MD patients. **E** Quantitative real-time PCR analysis of ISG in VEOs of VS and MD patients (*n* = 3 patients per group). **F** Representative immunofluorescence images showing STING (green), IBA1 (red) and DAPI (blue) staining in the ampullae and utricles of VS and MD patients (*n* = 3 patients per group, scale bar = 12·5 μm). **G** Images showing DHE staining in the BMs and ampullae of saline- or LPS-treated mice (*n* = 3 mice per group, scale bar = 100 μm). **H** Immune electron images showing mitochondrial (*) injury and dsDNA particles (yellow arrows) in hair cells of saline- or LPS-treated mice (*n* = 3 mice per group, scale bar = 500 nm). **I** Representative immunofluorescence images showing dsDNA (green) and Tomm20 (red) staining in the BMs and ampullae of saline- or LPS-treated mice (*n* = 3 mice per group, scale bar = 12·5 μm). **J** Western Blot to detect the expression of key proteins of the STING signaling pathway in the inner ear of saline- or LPS-treated mice. **K** Quantitative real-time PCR analysis of ISG in inner ear of saline- or LPS-treated mice (*n* = 3 mice per group). **L** Representative immunofluorescence images showing STING (green), IBA1 (red) and DAPI (blue) staining in the BMs and ampullae of saline- or LPS-treated mice (*n* = 3 mice per group, scale bar = 12·5 μm). BM, basilar membrane. Results are presented as mean ± SEM. *P < 0·05; **P < 0·01; ***P < 0·001, by analysis of two-tailed unpaired Student’s *t*-test

To investigate whether oxidative stress is involved in MD pathology, we evaluated ROS levels in VEOs using dihydroethidium (DHE) staining. Compared to VS patients, ROS levels of the VEOs were significantly increased in MD patients (Fig. 1A, Fig. S1B). Transmission electron microscope (TEM) examination revealed damaged mitochondria with decreased size and density, as well as reduced or absent mitochondrial cristae in sensory epithelial cells of MD patients’ VEOs (Fig. 1B). Furthermore, immunostaining of dsDNA and the mitochondrial marker Tomm20 distinguished the cytosolic DNA from nuclear or mitochondrial DNA, and it revealed a marked increase in cytosolic DNA in VEOs of MD patients (Fig. 1C, Fig. S1C). Meanwhile, IBA1^+^ macrophages were observed in close apposition with hair cells, whereas dsDNA signals appeared in extracellular space and partially overlapped with macrophages in MD (Fig. S1D).

Given that STING pathway functions as a cytosolic DNA sensing adaptor and results in the production of interferon-stimulated genes (ISG), we investigated whether STING signals were activated in MD. Western blot (WB) analyses showed that the expression and phosphorylation of STING, TBK1, and IRF3 were significantly upregulated in VEOs from MD patients compared with those from VS (Fig. 1D**)**. Our qRT-PCR data demonstrated a significant increase in mRNA levels of ISG, including *MX2*, *IL6*, *IFNB1*, *CXCL10*, *IFIT1*, *IFIT2*, *IFIT3* (Fig. 1E**)**. Collectively, these results suggest that significant oxidative stress and mitochondrial damage are present in VEOs of MD patients and are correlated with cytosolic DNA leakage and STING pathway activation. Confocal microscopy images revealed STING in VEOs with high expression being observed in IBA1-positive macrophages of MD patients (Fig. 1F, Fig. S1E). These results indicate that STING expression in macrophages was elevated significantly in VEOs of MD patients, potentially promoting further damage.

LPS-induced EH mouse model exhibited EH, hearing loss, and vestibular dysfunction, providing a valuable preclinical model for MD.^13^ DHE staining revealed an increased ROS level in basilar membranes and ampullae of LPS-treated mice (Fig. 1G, Fig. S1F). Co-immunostaining of dsDNA and Tomm20 was used to determine the presence of cytosolic DNA. Additionally, 10 nm colloidal gold particles marked small dsDNA particles outside nuclei and mitochondria, as we observed similar mitochondrial damage to that seen in MD patients, indicating cytoplasmic DNA leakage (Fig. 1H, I, Fig. S1G). Meanwhile, IBA1^+^ macrophages were observed in close apposition with both hearing and vestibular hair cells, and neighboring or overlapping released dsDNA (Fig. S1H). Furthermore, WB analysis revealed that the levels of STING, p-STING, TBK1, p-TBK1, IRF3 and p-IRF3 were elevated in the inner ear of LPS-treated mice (Fig. 1J). Additionally, the mRNA levels of ISG, including *Il6*,* Ifnb1*,* Ifit2*,* Ifit3*,* Mx1*,* Mx2*, *Usp18*, were also elevated (Fig. 1K). Immunofluorescence confirmed that STING co-localized with IBA1-positive macrophages, consistent with our findings in MD patients (Fig. 1L, Fig. S1I). These results suggest a potential link between sensory epithelial cell DNA leakage and macrophage STING pathway activation in both human and rodent models.

### The dsDNA secreted by oxidative stressed HEI-OC1 cells triggers macrophage STING pathway activation

**Fig. 2:**
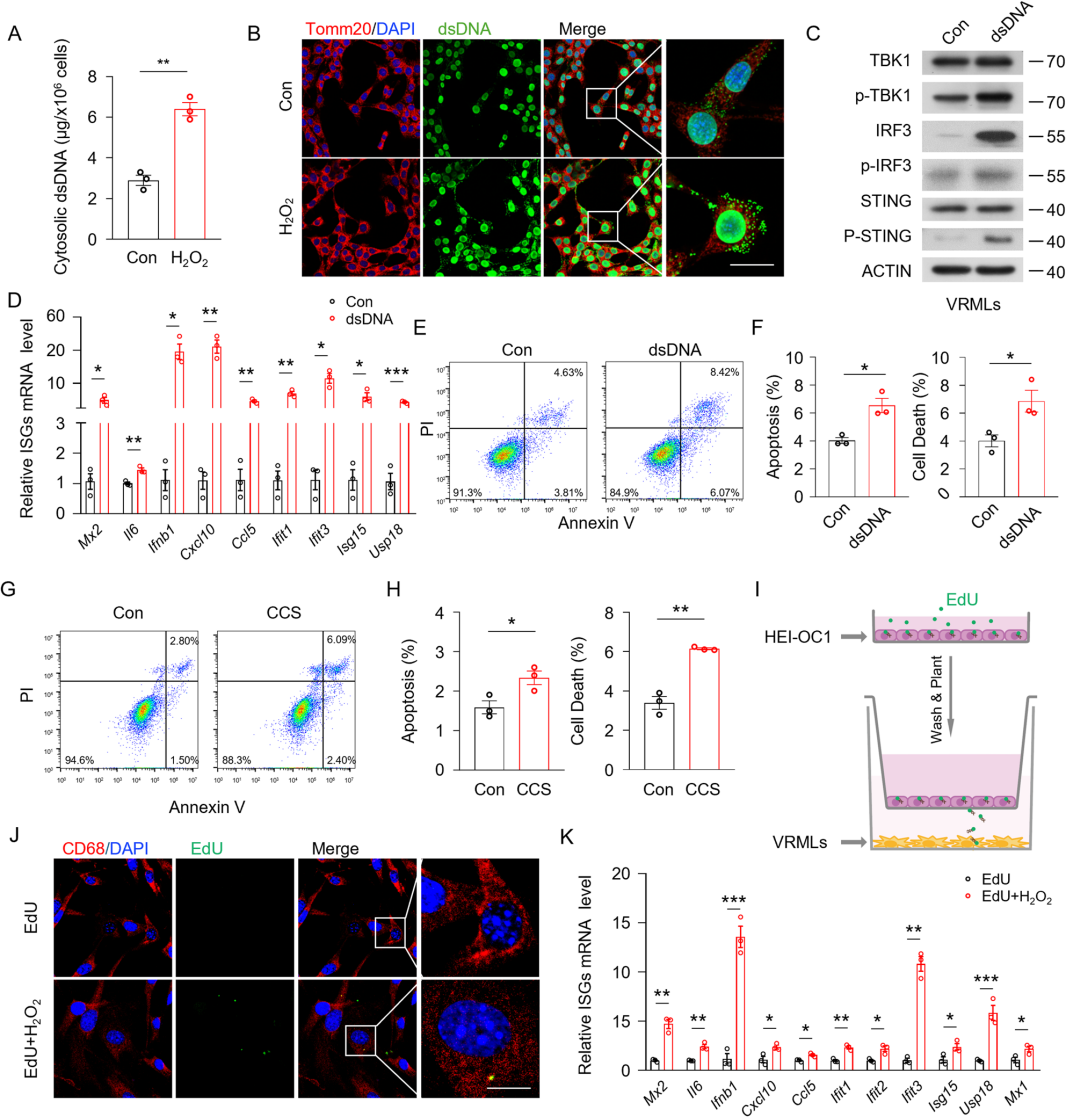
The dsDNA secreted by oxidative stressed HEI-OC1 cells triggers macrophage STING pathway activation. **A** Quantitative of dsDNA secretion culture supernatants from H_2_O_2_ stimulated or control HEI-OC1 cells (*n* = 3 biologically independent experiments). **B** Representative immunofluorescence images showing dsDNA (green), Tomm20 (red) and DAPI (blue) staining in H_2_O_2_ stimulated or control HEI-OC1 cells (*n* = 6 biologically independent experiments, scale bar = 25 μm). **C** Western Blot to detect the expression of key proteins of the STING signaling pathway in VRMLs that transfection with dsDNA from H_2_O_2_-treated HEI-OC1 cells (*n* = 3 biologically independent experiments). **D** Quantitative real-time PCR analysis of ISG in VRMLs that transfection with dsDNA from H_2_O_2_-treated HEI-OC1 cells (*n* = 3 biologically independent experiments). **E** Dot plot showing flow cytometry analysis VRML cells that transfected with dsDNA from H_2_O_2_-treated HEI-OC1 cells (*n* = 3 biologically independent experiments). **F** Analysis of flow cytometry of VRMLs that transfected with dsDNA from H_2_O_2_-treated HEI-OC1 cells (*n* = 3). **G** Dot plot showing flow cytometry analysis HEI-OC1 cells that are treated with CCS from dsDNA transfected VRMLs (*n* = 3 biologically independent experiments). **H** Analysis of flow cytometry of HEI-OC1 cells that treated with CCS from dsDNA transfected VRMLs (*n* = 3 biologically independent experiments). **I** Illustration of the cell treatment and transwell co-culture system. **J** Representative immunofluorescence images showing EdU (green), CD68 (red) and DAPI (blue) staining in VRMLs that co-cultured with EdU-labeled HEI-OC1 cells (*n* = 6 biologically independent experiments, scale bar = 12·5 μm). **K** Quantitative real-time PCR analysis of ISG in VRMLs that co-cultured with HEI-OC1 cells (*n* = 3 biologically independent experiments). VRMLs, vestibular-resident macrophage-like cells. CCS, cell culture supernatants. Results are presented as mean ± SEM. *P < 0·05; **P < 0·01; ***P < 0·001, by analysis of two-tailed unpaired Student’s *t*-test

To further determine whether oxidative stress exerts a direct role in DNA leakage in sensory epithelial cells, we treated HEI-OC1 cells with H_2_O_2_ in vitro. Upon H_2_O_2_ stimulation, the release of cytosolic dsDNA into the cell culture medium was significantly increased (Fig. 2A). Furthermore, an elevation in dsDNA levels was observed in the cytoplasm of HEI-OC1 cells (Fig. 2B, Fig.S2A). These results suggest that H_2_O_2_ stimulation induces dsDNA leakage and elevates the cytoplasmic dsDNA content.

Next, we isolated cytosolic dsDNA from H_2_O_2_-treated HEI-OC1 cells and performed dsDNA transfection in cultured VRML cells. Direct transfection of dsDNA significantly increased the phosphorylation of STING, TBK1, and IRF3 in VRML cells, indicating activation of this DNA sensing pathway (Fig. 2C). Additionally, the expression of ISG, including *Mx2*,* Il6*,* Ifnb1*,* Cxcl10*,* Cxcl5*,* Ifit1*,* Ifit3*,* Isg15*,* Usp18* was also elevated in dsDNA-treated VRML cells (Fig. 2D), leading to increased cell death and apoptosis (Fig. 2E, F). Conversely, cell culture supernatants (CCS) from dsDNA-stimulated VRML cells significantly increased cell death and apoptosis in HEI-OC1 cells (Fig. 2G, H). To verify the direct interaction between HEI-OC1 cells and VRML cells, a transwell co-culture system was employed (Fig. 2I). Specifically, HEI-OC1 cells were labeled with the DNA dye EdU, washed and placed in the upper chamber. VRML cells were seeded on the surface of the transwell chamber and co-cultured with HEI-OC1 cells, followed by treatment with H_2_O_2_. Confocal microscopy detected EdU-labeled DNA particles within the cytoplasm of VRML cells (Fig. 2J, Fig. S2B), confirming that VRML cells phagocytosed DNA from the injured HEI-OC1 cells. Additionally, the expression of ISG, including *Mx2*,* Il6*,* Ifnb1*,* Cxcl10*,* Cxcl5*,* Ifit1*,* Ifit2*,* Ifit3*,* Isg15*,* Usp18*,* Mx1*, was elevated in co-cultured VRML cells (Fig. 2K). To investigate whether cochlear and vestibular macrophages engulf exogenous dsDNA in vivo, we isolated EdU-labeled cytosolic dsDNA from H_2_O_2_-treated HEI-OC1 cells and microinjected it into the perilymph via the posterior semicircular canal (PSCC). As shown in Fig. S2C-E, EdU-labeled DNA particles were observed within macrophages. Collectively, these findings suggest that oxidative stress induces DNA damage in sensory epithelial cells, resulting in the release of dsDNA, which could be phagocytosed by macrophages and activate the STING signaling pathway, thereby influencing hair cell viability.

### *Sting* conditional deletion in macrophages ameliorates LPS-induced EH and audio-vestibular dysfunction

**Fig. 3:**
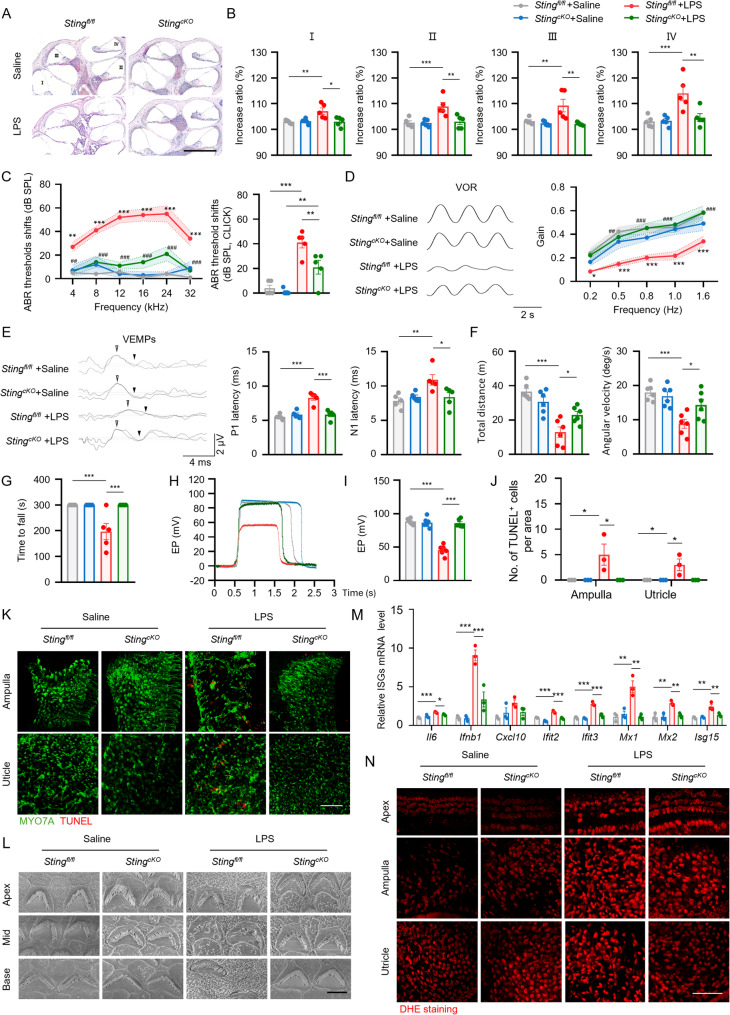
*Sting* conditional deletion in macrophages ameliorates LPS-induced EH and audio-vestibular dysfunction. **A** Representative images of mid-modiolar cochlear sections, scale bar = 200 μm. **B** Measurements of IR-L in cochlear half-turns I–IV (*n* = 5 mice per group). **C** ABR threshold shifts of *Sting*^*fl/fl*^ and *Sting*^*cKO*^ mice after saline or LPS treated (*n* = 5 mice per group, *Sting^*fl/fl*^ +saline group compared to *Sting*^*fl/fl*^ +LPS group, # *Sting*^*fl/fl*^ +LPS group compared to *Sting*^*cKO*^ +LPS group). **D** Representative horizontal VOR waves and VOR gains of all groups at a peak velocity (20^◦^/s) and frequencies range from 0·2 to 1·6 Hz (*n* = 5 mice per group, * *Sting*^*fl/fl*^ +saline group compared to *Sting*^*fl/fl*^ +LPS group, # *Sting*^*fl/fl*^ +LPS group compared to *Sting*^*cKO*^ +LPS group). **E** Representative click-evoked VEMPs waves and the P1 (white triangle) and N1 (black triangle) peak latencies of VEMPs (*n* = 5 mice per group). **F** Quantification of total distance and angular velocity in open field tests (*n* = 6 mice per group). **G** Quantification of rotarod test (*n* = 5 mice per group). **H**,** I** EP recorded from the scala media of saline or LPS treated *Sting*^*fl/fl*^ and *Sting*^*cKO*^ mice (*n* = 6 mice per group). **J** Quantification of the number of TUNEL+ cells per area in the ampullae and utricles of saline or LPS treated *Stingfl/fl* and *StingcKO* mice (*n* = 3 mice per group, scale bar = 50 μm). ROIs were defined as 150 μm square. **K** Representative immunofluorescence images showing MYO7A (green) and TUNEL (red) staining in the ampullae and utricles of saline or LPS treated *Stingfl/fl* and *StingcKO* mice (*n* = 3 mice per group, scale bar = 50 μm). **L** Electron microscopy images showing stereocilia bundles in OHC of saline or LPS treated *Sting*^*fl/fl*^ and *Sting*^*cKO*^ mice (*n* = 3 mice per group, scale bar = 3 μm). **M** Quantitative real-time PCR analysis of ISG in inner ear of saline or LPS treated *Sting*^*fl/fl*^ and *Sting*^*cKO*^ mice (*n* = 3 mice per group). **N** Images showing DHE staining in the vitro-cultured tissues (*n* = 3 mice per group, scale bar = 50 μm). EP, endocochlear potential. Results are presented as mean ± SEM. *P < 0·05; **P < 0·01; ***P < 0·001, by analysis of one-way ANOVA followed by Tukey’s test (**B**, **C**, **D**, **F**, **G**, **I**, **J**, **M**) and two-way ANOVA followed by Fisher’s LSD post hoc test (**C**, **D**)

Next, we generated myeloid cell-specific deletion of *Sting* gene (*Sting*^*cKO*^) mice (Fig. S3A) and administered LPS into both *Sting*^*cKO*^ and littermate *Lyz2-Cre*^*−/−*^;*Sting*^*flox/flox*^ control (*Sting*^*fl/fl*^) mice to investigate the role of STING in macrophages in LPS-induced audio-vestibular dysfunction. Body weights of both groups were monitored to assess their overall health and systemic responses to the LPS administration (Fig. S3B). Subsequently, the temporal bones of these mice were harvested for quantitative assessment of changes in endolymphatic space in cochlea to evaluate the extent of EH (Fig. 3A). LPS administration induced significant EH in *Sting*^*fl/fl*^ mice but not in *Sting*^*cKO*^ mice, indicating that STING in macrophages is critical in the development of EH (Fig. 3B).

To evaluate auditory function in LPS-treated mice, we assessed the click- and tone-burst-evoked ABR thresholds before and after LPS injection. Compared to *Sting*^*fl/fl*^ mice, LPS-induced ABR threshold shifts were significantly reduced in *Sting*^*cKO*^ mice (Fig. 3C). Similarly, DPOAE amplitudes were significantly reduced in LPS-treated *Sting*^*fl/fl*^ mice, but were preserved in *Sting*^*cKO*^ (Fig. S3C, D). We further performed VOR, VEMPs, rotarod test and open field test to evaluate vestibular function of mice. LPS-treated *Sting*^*fl/fl*^ mice exhibited decreased horizontal VOR gain at 20°/s across all tested stimulus frequencies (0·2, 0·5, 0·8, 1·0, and 1·6 Hz) (Fig. 3D). The positive peak (P1) and negative peak (N1) latencies in VEMPs were prolonged in LPS-treated *Sting*^*fl/fl*^ mice, whereas *Sting*^*cKO*^ mice had no significant abnormalities (Fig. 3E, Fig. S3E). In behavioral testing, *Sting*^*cKO*^ mice exhibited increased activation and angular velocity in the open field test (Fig. 3F, Fig. S3F**)**, and stayed for a longer time in accelerating rotarod (Fig. 3G). Together, these findings suggest that *Sting*^*cKO*^ mice exhibited milder audio-vestibular dysfunction following LPS challenge compared to *Sting*^*fl/fl*^ mice.

To further elucidate the underlying mechanisms of the observed auditory dysfunction, we examined EP in LPS-treated *Sting*^*fl/fl*^ and *Sting*^*cKO*^ mice. The positive EP was significantly reduced (88.35 ± 1.79 mV vs. 45.75 ± 3.1 mV, *p* < 0.001) in LPS-treated *Sting*^*fl/fl*^ mice, while *Sting*^*cKO*^ mice maintained near normal EP values under saline or LPS treatment (Fig. 3H, I). Since EP is generated by the cochlear lateral wall, we measured SV thickness and observed a significant reduction in LPS-treated *Sting*^*fl/fl*^ mice, but observed a preservation SV morphology in LPS-treated *Sting*^*cKO*^ mice. SLi fibrocyte density showed no significant changes (Fig. S4A, B). These findings suggest that macrophage-specific *Sting* deletion protects ion homeostasis and hair cell sensory transduction in LPS-treated mice. The ampullae and utricles of *Sting*^*fl/fl*^ mice had a higher number of TUNEL-positive vestibular hair cells following LPS challenge compared to *Sting*^*cKO*^ mice (Fig. 3J, K**)**. Similarly, LPS challenge resulted in a significant induction of TUNEL-positive signals in both outer hair cells (OHC) and inner hair cells (IHC) in the apical turn of *Sting*^*fl/fl*^ mice compared to *Sting*^*cKO*^ mice, while no significant differences were observed in middle and basal turn (Fig. S4C, D). Quantification of hair cell numbers confirmed that *Sting*^*fl/fl*^ mice exhibited marked OHC loss in the apical, middle and basal turn after LPS treatment, but found no significant IHC loss (Fig. S4E, F). Furthermore, SEM revealed that OHC of LPS-treated *Sting*^*fl/fl*^ mice exhibited stereocilia fusion, bundle distortion, and individual stereocilia breakage, whereas stereocilia morphology was intact in *Sting*^*cKO*^ mice (Fig. 3L). Additionally, loss of ribbon synapses reduced the number of auditory nerve fibers responsive to sound. Immunofluorescence staining of ribbon synapses using anti-CtBP2 antibodies further demonstrated that ribbon synapse count diminished in LPS-treated *Sting*^*fl/fl*^ mice, but was maintained at a higher level in LPS-treated *Sting*^*cKO*^ mice (Fig. S4G, H). Collectively, LPS-induced hair cell damage primarily affected OHCs, the impairment resulted from a reduced EP by decreasing the transduction driving force, along with damaged stereocilia that disrupted the cochlear amplification process.

We further confirmed that expression of ISG, including *Il6*,* Ifnb1*,* Cxcl10*,* Cxcl5*,* Ifit2*,* Ifit3*,* Mx1*,* Mx2*, *Isg15*, was upregulated in LPS-treated *Sting*^*fl/fl*^ mice, and reduced in LPS-treated *Sting*^*cKO*^ mice (Fig. 3M). After LPS treatment, IBA1^+^ macrophages increased in the basilar membranes and SV in both *Sting*^*fl/fl*^ and *Sting*^*cKO*^ mice (Fig. S4I, J). Furthermore, DHE staining were performed in saline or LPS-stimulated vitro-cultured tissues, revealing enhanced ROS production in *Sting*^*fl/fl*^ mouse tissues, which was markedly reduced in *Sting*^*cKO*^ mouse tissues (Fig. 3N). Collectively, these findings underscore that macrophage-specific STING acts as a critical upstream trigger for LPS-induced audio-vestibular dysfunction.

### CRL4B mediates K48-linked ubiquitination of STING in K370 and regulates STING stability

**Fig. 4:**
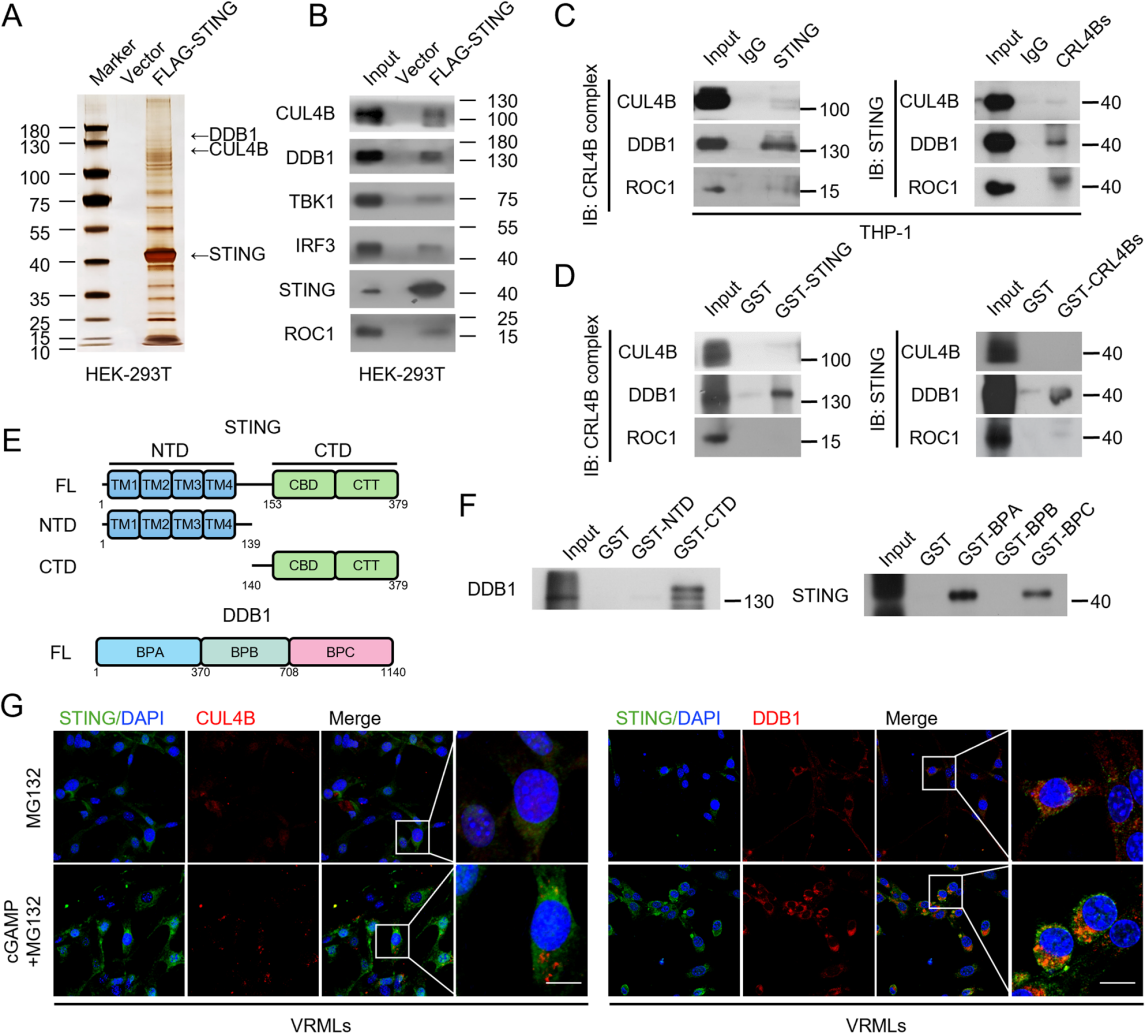
STING co-localizes and interacts with CRL4B complex. **A** Immunoaffinity purification and mass spectrometry analysis of a protein complex containing the STING protein in HEK-293T cells. SDS-PAGE and silver-staining were performed to exhibit STING co-purified protein. **B** Western blot analysis of the purified fractions of key proteins in HEK-293T cells. **C** Co-IP analysis of the interaction between STING and CRL4Bs in THP-1 cells. **D** GST pull-down assay with STING and CRL4Bs. **E** Schematic diagram annotation of the essential domains of STING and DDB1 required for interaction. **F** GST pull-down assay identifies the essential STING domains required for the interaction with DDB1 and the essential DDB1 domains required for the interaction with STING. **G** Representative immunofluorescence images showing STING (green), CUL4B/DDB1 (red) and DAPI (blue) staining in VRMLs treaded with cGAMP and/or MG132 (*n* = 3 biologically independent experiments, scale bar = 12·5 μm). VRMLs, vestibular-resident macrophage-like cells

Expression and post-translational modification of STING is essential in various biological processes, highlighting the need to clarify the mechanisms that regulate its protein levels [33, 34]. We further performed immunoprecipitation-mass spectrometry (IP-MS) analysis to identify associated proteins and investigate the regulatory mechanisms underlying STING activation in MD. Figure 4A showed that STING was co-purified with various proteins, and the source data is available in Data S1. Notably, the presence of CUL4B and DDB1 suggests that STING may physically interact with and potentially undergo modification by the CRL4B complex.

The presence of these proteins was verified using WB, along with TBK1 and IRF3, which function together with STING as downstream components of the pathway (Fig. 4B). To investigate the interaction between CRL4B complex and STING, we performed Co-IP assays in macrophage cell lines and found that STING co-immunoprecipitated with the CRL4B complex, primarily with DDB1 (Fig. 4C, Fig. S5A). GST pull-down assay indicated that STING directly interacted with DDB1 (Fig. 4D), consistent with the previous research findings that DDB1 functioned as substrate-binding protein in CRL4B complex [35]. STING contains two domains, the N-terminal (NTD) contains four transmembrane regions (TM) and anchors the protein to the ER membrane, and the C-terminal (CTD) contains the cyclic dinucleotide-binding domain (CBD) and the C-terminal tail (CTT). DDB1 contains three β propeller (BP) domains, BPA and BPB form a pocket for potential interaction (Fig. 4E). GST-pull down assay demonstrated that STING CTD directly binds with DDB1, and BPA and BPC of DDB1 are responsible for STING-DDB1 interaction (Fig. 4F).

**Fig. 5:**
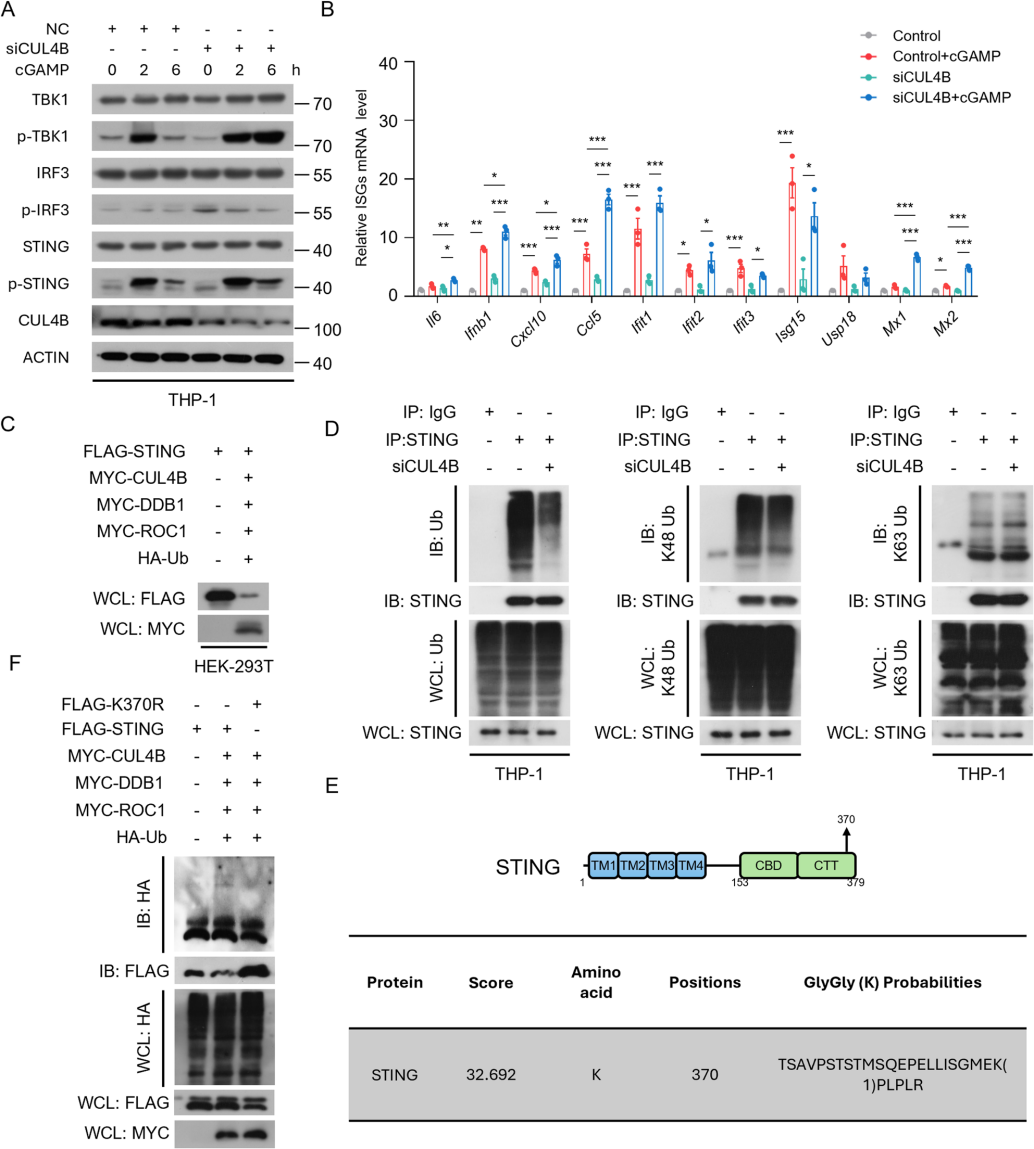
CUL4B mediates the ubiquitination and degradation of STING. **A** Western Blot to detect the expression of key proteins of the STING signaling pathway in THP-1 cells that transfection with or without siCUL4B and treated with cGAMP. **B** Quantitative real-time PCR of indicated ISG of Control and CUL4B siRNA-transfected THP-1 cells and treated with cGAMP (2 h, *n* = 3 biologically independent experiments). **C** Western blot analysis of the whole cell lysate of HEK-293T cells with or without the absence and presence of MYC-CRL4Bs, FLAG-STING and HA-Ub. **D** Co-IP analysis of STING ubiquitination in THP-1 cells that transfection with or without siCUL4B. **E** IP-MS/MS to identify the specific ubiquitination site of STING. **F** Co-IP analysis of ubiquitination of FLAG-STING or FLAG-K370R with or without the presence of MYC-CRL4Bs and HA-Ub with MG132 (4 h). Results are presented as mean ± SEM. *P < 0·05; **P < 0·01; ***P < 0·001, by analysis of one-way ANOVA followed by Tukey ‘s test

Consistently, immunofluorescence assays showed CUL4B and DDB1 co-localized with STING within VRML cells under cGAMP stimulation and MG132 treatment (Fig. 4G), suggesting that CRL4B may be involved in the regulation of STING in macrophages. We knocked down CUL4B in THP-1 cells and detected the activation of STING, TBK1, and IRF3. The phosphorylation levels of these proteins were upregulated in CUL4B-deficient cells under cGAMP and MG132 treatment (Fig. 5A). ISG mRNA expression was significantly higher after treatment with siCUL4B than siNC (Fig. 5B). Additionally, we co-expressed MYC-CUL4B, MYC-DDB1, MYC-ROC1, FLAG-STING and HA-Ub, without the treatment of MG132, STING expression was significantly decreased compared with the vector (Fig. 5C).

Considering the strong regulatory control over STING’s function and stability, we examined whether STING could act as a ubiquitination substrate of the CRL4B complex. As shown in Fig. 5D, the total ubiquitination level and K48-linked ubiquitination level of STING, but not K63-linked ubiquitination level, were decreased in CUL4B-deficient cells. STING contained multiple lysine (K) residues that can bind to ubiquitin. We used LC-MS/MS to identify the specific ubiquitination site of STING. The results demonstrated that Lys residue K370 of STING exhibited ubiquitin-modification signals (Fig. 5E, Data S2). Consequently, we mutated K370 into Arg in plasmids (FLAG-K370R). Compared with FLAG-STING, the ubiquitination of FLAG-K370R showed a significant decrease in the presence of MYC-CRL4Bs and HA-Ub (Fig. 5F). These results indicate that CRL4B complex binds to STING through substrate binding protein DDB1 and promotes K48-linked ubiquitination at K370.

### The effect of CUL4B on LPS-induced damage relied on STING in double conditional knockout mice

**Fig. 6:**
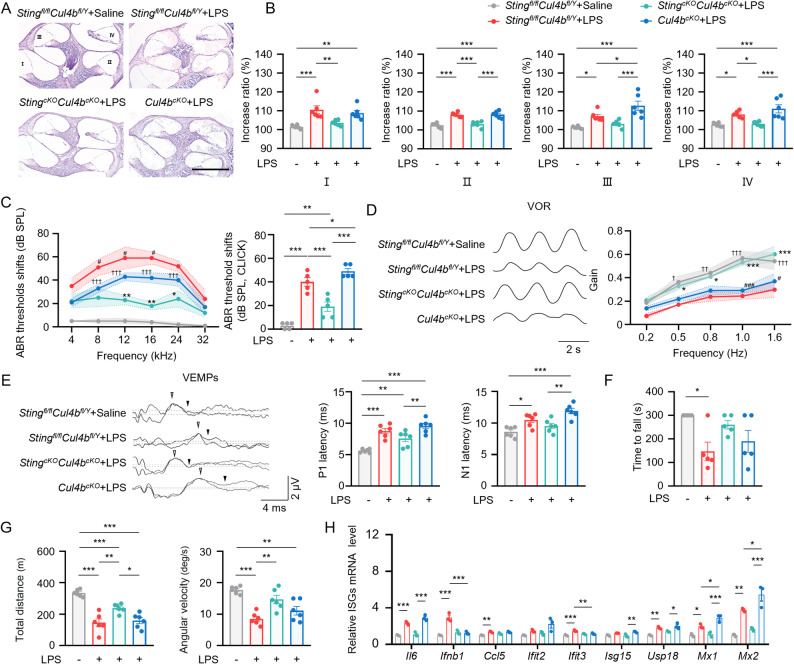
The effect of *Cul4b* on LPS-induced damage relied on *Sting* in double conditional knockout mice. **A** Representative images of mid-modiolar cochlear sections, scale bar = 200 μm. **B** Measurements of IR-L in cochlear half-turns I–IV (*n* = 6 mice per group). **C** ABR threshold shifts of *Sting*^*fl/fl*^*Cul4b*^*fl/Y*^, *Cul4b*^*cKO*^ and *Sting*^*cKO*^*Cul4b*^*cKO*^ mice after saline or LPS treated (*n* = 5 mice per group). **D** Representative horizontal VOR waves and VOR gains of all groups (*n* = 5 mice per group, ). **C**,** D** # *Cul4b*^*cKO*^+LPS group compared to *Sting*^*fl/fl*^*Cul4b*^*fl/Y*^+LPS group, * *Sting*^*fl/fl*^*Cul4b*^*fl/Y*^+LPS group compared to *Sting*^*cKO*^*Cul4b*^*cKO*^+LPS group, ^†^*Sting*^*cKO*^*Cul4b*^*cKO*^+LPS group compared to *Cul4b*^*cKO*^+LPS group. **E** Representative click-evoked VEMPs waves, the P1 (white triangle) and N1 (black triangle) peak latencies of VEMPs at 100 dB nHL (*n* = 6 mice per group). **F** Quantification of rotarod test (*n* = 5 mice per group). **G** Quantification of total distance and angular velocity in open field tests (*n* = 6 mice per group). **H** Quantitative real-time PCR analysis of ISG in inner ear of saline or LPS treated *Sting*^*fl/fl*^*Cul4b*^*flY*^, *Cul4b*^*cKO*^ and *Sting*^*cKO*^*Cul4b*^*cKO*^ mice (*n* = 3 mice per group). Results are presented as mean ± SEM. *P < 0·05; **P < 0·01; ***P < 0·001, by analysis of one-way ANOVA followed by Tukey’s test (B, C, E, F, G, H) and two-way ANOVA followed by Fisher’s LSD post hoc test (C, E)

To explore whether CUL4B influences STING-mediated responses in vivo through ubiquitination-dependent degradation, we generated *Cul4b*^*cKO*^ mice and double conditional knockout (*Sting*^*cKO*^*Cul4b*^*cKO*^) mice. LPS induced severe EH in *Sting*^*fl/fl*^*Cul4b*^*fl/Y*^ mice, while *Sting*^*cKO*^*Cul4b*^*cKO*^ mice showed no significant EH after LPS treatment (Fig. 6A, B, Fig. S5B).

Furthermore, in *Cul4b*^*cKO*^ mice, LPS-induced ABR threshold shifts were significantly increased compared with *Sting*^*fl/fl*^*Cul4b*^*fl/Y*^ mice, while in *Sting*^*cKO*^*Cul4b*^*cKO*^ mice, these shifts were reduced (Fig. 6C). LPS-treated *Sting*^*fl/fl*^*Cul4b*^*fl/Y*^ mice exhibited decreased vestibular function (Fig. 6D-G, Fig. S5C, D). *Cul4b*^*cKO*^ mice demonstrated a significantly reduced horizontal VOR gain at 20°/s in 1·0, and 1·6 Hz (Fig. 6D) and prolonged P1 and N1 VEMP peak latency (Fig. 6E), indicating worsened vestibular dysfunction. This effect was reversed in *Sting*^*cKO*^*Cul4b*^*cKO*^ mice. Additionally, *Ifnb1*,* Ifit3*,* Mx1*, and *Mx2* were upregulated in LPS-treated *Cul4b*^*cKO*^ mice, but not in *Sting*^*cKO*^*Cul4b*^*cKO*^ mice (Fig. 6H). These findings indicate that CUL4B deficiency plays a role in ameliorating audio-vestibular dysfunction, likely through modulation of the STING signaling pathway.

## Discussion

MD is a representative vestibular disorder, yet the immunopathological mechanisms underlying its vestibular dysfunction remain poorly understood [36, 37]. In this study, we found that significant oxidative stress and mitochondrial damage occur in vestibular tissues from both MD patients and LPS-induced EH mouse models, correlating with cytosolic DNA leakage and subsequent STING pathway activation. Using an in *vitro* co-culture system, we found that the STING signaling in VRML cells activated by dsDNA released from sensory epithelial cells contributes to a positive feedback loop that exacerbates damage to sensory epithelial cells. In *vivo*, macrophage-specific STING deletion significantly alleviates LPS-induced audio-vestibular damage. STING interacts with CRL4B complex via DDB1 and undergoes K48-linked ubiquitination. Then, we demonstrated that CUL4B regulates STING activity to influence LPS-induced damage using *Cul4b* and *Sting* double conditional knockout mice. Therefore, STING activation in vestibular macrophages contributes to audio-vestibular dysfunction in MD, and targeting STING has therapeutic implications for MD.

MD is primarily characterized by EH, an abnormal fluid accumulation in the inner ear, which is associated with increased intracochlear pressure and distortion of inner ear structures. The elevated pressure can disrupt cellular function and has been linked to oxidative stress. Previous studies have reported elevated indices of oxidative stress in MD, including upregulation of inducible nitric oxide synthase (iNOS) in the macula utricles and increased protein carbonyls in peripheral lymphocytes [25, 26]. Our findings corroborate these observations, as we detected markedly elevated ROS level in the VEOs of MD patients, accompanied by cytoplasmic DNA fragmentation and mitochondrial damage in hair cells. The dsDNA accumulates in the cytoplasm and potently activates the STING signaling pathway within resident macrophages [38]. This process not only involves the response of macrophages, but also the interaction between cells plays an important role in this process [39–41]. Our co-culture experiments further elucidate this process, demonstrating that dsDNA derived from sensory epithelial cells under oxidative stress can be engulfed by VRML cells, leading to enhanced STING activation. This intercellular communication axis underscores the importance of cell-cell interactions in elicitation and amplifying inflammation. Unveiling this mechanism aligns with existing literature on other pathological conditions, such as Parkinson’s disease [40], systemic sclerosis (SS) [22], acute lung injury (AKI) [42], and osteoarthritis [43] where oxidative stress-induced cytosolic DNA leakage similarly triggers innate immune responses through the cGAS-STING pathway. These findings collectively suggest that oxidative stress-mediated DNA damage activates the STING pathway, contributing to immune dysregulation critical and playing a critical role in MD pathogenesis.

Beyond their classical antiviral functions, dysregulated type I IFNs have been increasingly implicated in autoimmune and autoinflammatory disorders [44]. For instance, IFI44L, ISG15, and ITGB2 are relevant in SS and arthritis [45]. Likewise, dysregulated type I IFN–ISG pathways drive inflammation in systemic lupus erythematosus **(**SLE) and psoriasis via TLRs [46]. Numerous studies have consistently demonstrated elevated levels of pro-inflammatory cytokines, particularly those within the interferon pathway, in both the peripheral blood and the inner ear of MD patients. In PBMCs, the upregulation of specific cytokines such as *IL-6*, *IL-1β*, and *INHBA* has been used to define a proinflammatory subgroup of MD, underscoring the systemic involvement of inflammation in the disease [47]. Another study has confirmed the elevated expression of IFN-γ, IL-6, and TNF-α in ES luminal fluid, and also confirmed that a local inflammatory response underlies the mechanism of MD patients [48]. Our findings reveal an increase in STING phosphorylation activation, accompanied by a notable elevation in ISG expression, suggesting a potential regulatory role of the STING pathway in disease progression through ISG expression. While the downstream STING–ISG axis is a broadly conserved inflammatory pathway across tissues, our data highlight its relevance in the inner-ear milieu by connecting STING activation/ISG induction with sensory epithelial injury phenotypes in MD. IFNs tend to induce a strong pro-inflammatory response in sensory epithelial cells, which in turn cause functional damage. For example, IFN-γ directly induces retinal pigment epithelium cell death through ferroptosis via the JAK/STAT pathway [49]. Type I IFNs selectively induced an IRF1-dependent inflammatory immune response, promoting inflammatory responses and contributing to tissue damage [50]. Similarly, in vitro, we found that hair cell damage after being stimulated by CCS from macrophages, which were treated by dsDNA. Although the specific mechanism remains under investigation, the role of STING/ISG signaling in driving sensory epithelial cells damage is a key area of research focus.

Post-translational modifications of STING are essential for either activation or degradation, crucial in immune regulation and represents a potential therapeutic target for sterile inflammatory diseases [51]. In this study, we identified that CRL4B complex interacts with STING via the adaptor molecule DDB1 and mediates K48-linked polyubiquitination at K370 residues, leading to STING degradation. It is reported that K11-linked, K48-linked, K63-linked and other ubiquitination significantly impact STING activation, trafficking, and signaling pathways. Notably, K48-linked ubiquitination promoted by TRIM13, TRIM27, or RNF5 predominantly targets STING for proteasomal degradation, thereby negatively regulating inflammatory signaling pathways [52–54]. Adding to these important findings, our data suggest that CUL4B also targets STING for K48-linked ubiquitination and degradation. A recent study similarly reported AhR-dependent K48-linked ubiquitination of STING at the K236 site, exerting immunosuppressive effects in bladder cancer [55]; however, our findings demonstrate that the CUL4B–DDB1 complex mediates STING ubiquitination and degradation at a distinct residue, K370, suggesting that CUL4B may exert anti-inflammatory functions across various diseases by targeting distinct STING ubiquitination sites in a tissue-specific manner. In addition, studies have shown that CUL4B fostering a balance between TH1 and TH2 subsets [56], promoting macrophage migration and adhesion in diabetic kidney disease [57], and regulating TLR-triggered inflammation during infection [58]. Our findings revealed that specific deletion of *Cul4b* in macrophages exacerbates inflammation and audio-vestibular dysfunction in LPS-induced mouse models. Furthermore, the rescue of the exacerbated phenotype resulting from double genetic deletion of *Sting* and *Cul4b* further validates that CUL4B exerts its anti-inflammatory effects through STING. Collectively, these findings highlight the pivotal role of CUL4B as a braking factor in the downregulation of STING-related immune responses, emphasizing the importance of exploring its influence in advancing our understanding and development of therapies for MD.

By inhibiting the abnormally activated STING signaling pathway, STING inhibitors have been shown to ameliorate LPS-induced acute lung injury, suppress the expression of inflammatory cytokines and attenuate pathological changes in lung tissue [59]. Recent studies suggest that inhibition of STING pathway disrupts the lupus phenotypes through its effect on dendritic cells differentiation [60]. *Sting* knockout in macrophages significantly protects audio-vestibular function, highlighting the crucial role of the STING pathway in MD occurrence. While the development of STING inhibitors remains in its early stages, STING-specific inhibitors (SN-011, C-176, 4-OI and LB244) have shown significant therapeutic promise for a range of inflammatory and autoimmune diseases [61–64]. Thus, targeting the STING-ISG axis represents a promising therapeutic approach for controlling sterile inflammation and mitigating disease progression in MD. Future studies should focus on profiling ISG transcripts in MD patient tissues, correlating their expression with clinical disease markers, and exploring therapeutic modulation of these pathways to identify novel intervention strategies.

Our results collectively demonstrate that LPS-induced ABR threshold elevation arises from STING-mediated cochlear inflammation acting through multiple structural lesions, among which EP reduction and hair cell pathology represent the primary contributors. Specifically, our findings reveal a significant reduction in the EP in LPS-treated *Sting*^*fl/fl*^ mice, which provides the driving force for mechanoelectrical transduction in hair cells [65–67]. Meanwhile, we observed a reduction in SV thickness, which may lead to an imbalance in ion homeostasis and a subsequent reduction in the EP. Concurrently, the marked OHC loss and stereocilia disorganization reduced DPOAE amplitudes in LPS-treated *Sting*^*fl/fl*^ mice, reflecting an impaired cochlear amplifier [68–70]. Notably, the increased apoptosis revealed by TUNEL staining was most pronounced in the apical turn. This spatial pattern of cell death may provide mechanistic insights into the pathogenesis of low-frequency sensorineural hearing loss associated with MD. The observed cochlear synaptopathy in LPS treated *Sting*^*fl/fl*^ mice has a minor contribution to ABR threshold elevation as the reduced number of auditory nerve fibers activated may affect ABR readout, particularly by reducing signal to noise ratio at near threshold levels [71]. Localized cochlear inflammation acts as the upstream trigger, promoting pro-inflammatory cytokine release and subsequent cellular injury. However, potential contributors, such as spiral ganglion neuron damage and specific ionic imbalances, remain to be further investigated.

While our findings provide mechanistic insights into MD immunopathology, several limitations merit consideration. Due to the difficulties in obtaining normal human vestibular tissue, researchers commonly used vestibular tissue from patients with VS who underwent labyrinthectomy as a control group [72–74]. VS, or acoustic neuroma, are benign tumours originating from the Schwann cells, located away from the vestibular tissue and largely affect the nerve rather than the inner ear structures. Ishiyama et al. reported that the VEOs in MD show significant structural changes not usually found in VS, which tends to retain more normal histological features [75]. Nevertheless, as a neoplastic condition, VS may introduce potential tumour-associated inflammatory confounders in molecular comparisons and should be considered. Additionally, the EH mouse model induced by short-term LPS administration, which primarily reflects acute inflammatory changes and does not fully recapitulate the chronic, fluctuating, and idiopathic nature of MD. While the model is useful for mechanistic investigation, caution is needed when extrapolating these findings to the long-term clinical course of MD, and further studies using chronic or complementary models will be necessary.

Overall, our findings underscore the critical role of STING-mediated inflammation in MD pathology, providing new insights into the mechanisms that connect sensory epithelial damage and macrophage-driven inflammation. The identification of the CRL4B complex as an intrinsic regulator of STING signaling enhances our understanding of the inflammatory cascade in MD and highlights the therapeutic potential of targeting STING or its regulatory machinery to disrupt the feedback loops.

## Supplementary Information

Supplementary Material 1.

Supplementary Material 2.

Supplementary Material 3.

## Data Availability

All data needed to evaluate the conclusions in the paper are present in the paper and/or the Supplementary Materials. Materials and associated protocols are available upon request from the corresponding authors.
