# Supplementary material for: STING signaling in vestibular macrophages underlies Ménière’s disease pathogenesis
Source: J Neuroinflammation. 2026 Apr 14;23:161. doi: 10.1186/s12974-026-03812-4 (PMC13200447; doi:10.1186/s12974-026-03812-4)
Supplement: Supplementary file 1 — Supplementary Material 1. [file 12974_2026_3812_MOESM1_ESM.docx]

Supplementary Materials for

# STING Signaling in Vestibular Macrophages underlies Ménière’s Disease pathogenesis

Jiahui Liu *et al.*

*Corresponding authors: whboto11@email.sdu.edu.cn (H.W.).

zhangdaogong1978@163.com (D.Z), linda@email.sdu.edu.cn (N.L.).

**This file includes:**

Figs. S1 to S5

Tables S1 to S2

**Other Supplementary Materials for this manuscript include the following:**

Data S1 to S2


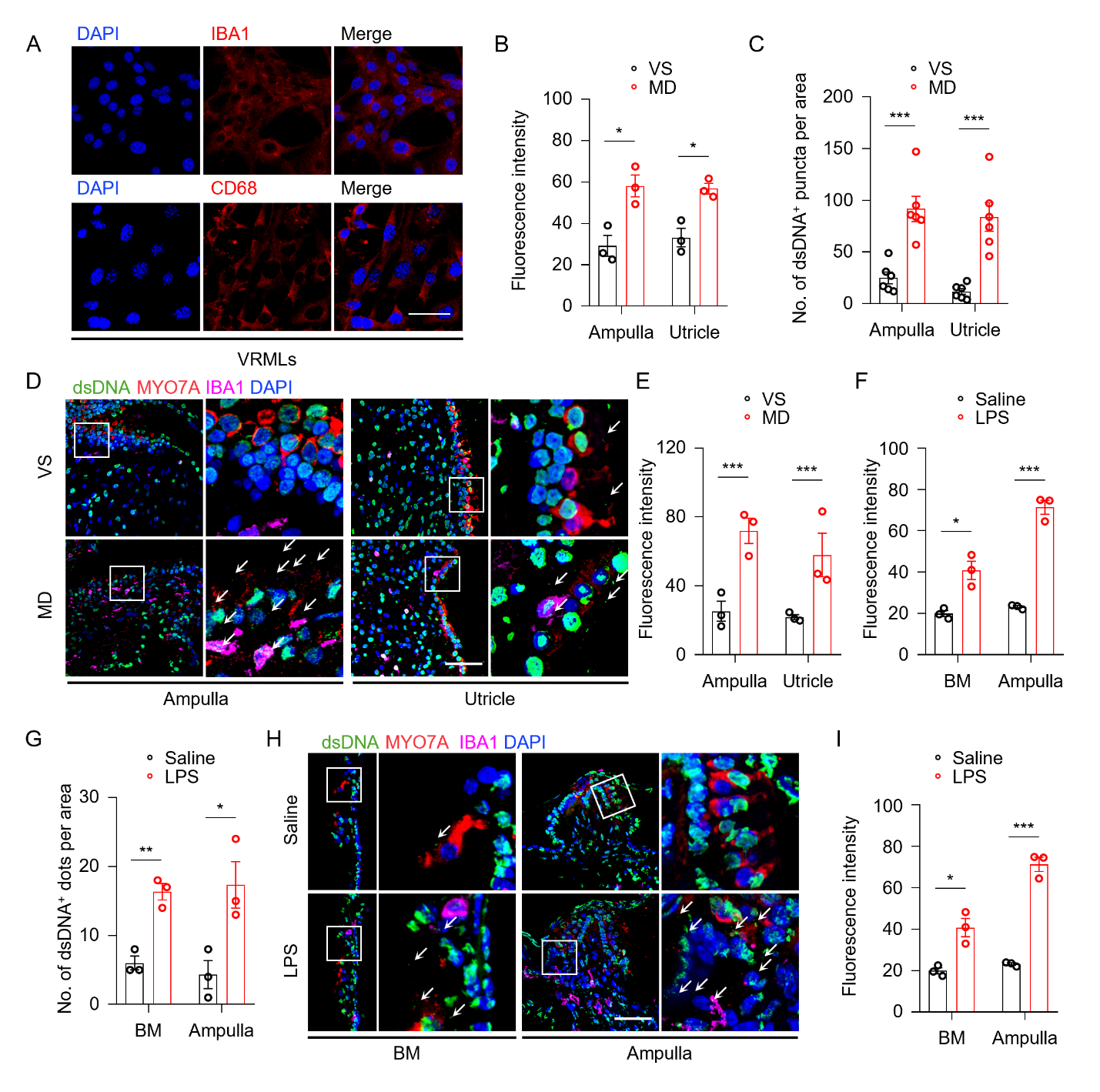


Fig. S1 Sensory epithelial cell DNA leakage and STING signaling activation in VEOs of MD patients and inner ear of LPS-induced EH mice model.

**(A)** Representative immunofluorescence images showing IBA1 or CD68 (red), and DAPI (blue) staining in the VRMLs (n = 3 biologically independent experiments). **(B)** Quantification of DHE staining intensity in the ampullae and utricles of VS and MD patients (n = 3 patients per group). **(C)** Quantification of cytosolic dsDNA^+^ puncta in the ampullae and utricles of VS and MD patients (n = 6 patients per group). ROIs were defined around hair cells in tissue sections as 100 µm by 50 µm areas. **(D)** Representative immunofluorescence images showing dsDNA (green), MYO7A (red), IBA1 (magenta) and DAPI (blue) staining in the ampulla**e** and utricles of VS and MD patients (n = 6 patients per group, scale bar = 50 μm). **(E)** Quantification of STING staining intensity in ampullae and utricles of VS and MD patients (n = 3 patients per group). ROIs were defined as the area of IBA1^+^ red staining. (**F**) Quantification of DHE staining intensity in the BMs and ampullae of saline- or LPS-treated mice (n = 3 mice per group). ROIs were defined around hair cells in tissue sections as 100 µm by 50 µm areas. (**G**) Quantification of dsDNA^+^ dots in the BMs and ampullae of saline- or LPS-treated mice (n = 3 mice per group). ROIs were defined around hair cells in tissue sections as 100 µm by 50 µm areas. **(H)** Representative immunofluorescence images showing dsDNA (green), MYO7A (red), IBA1 (magenta) and DAPI (blue) staining in the BMs and ampullae of saline- or LPS-treated mice (n = 3 mice per group, scale bar = 50 μm). **(I)** Quantification of STING staining intensity of the BMs and ampullae of saline- or LPS-treated mice (n = 3 mice per group). **(D, H)** The right panels represent 4-fold enlargements of the white-boxed areas in the left panels. White arrows indicate dsDNA^+^ puncta. ROIs were defined as the area of IBA1^+^ red staining. BM, basilar membrane. VRMLs, vestibular-resident macrophage-like cells. Results are presented as mean ± SEM. **P* < 0·05; ***P* < 0·01; ****P* < 0·001, by analysis of two-tailed unpaired Student's *t*-test.


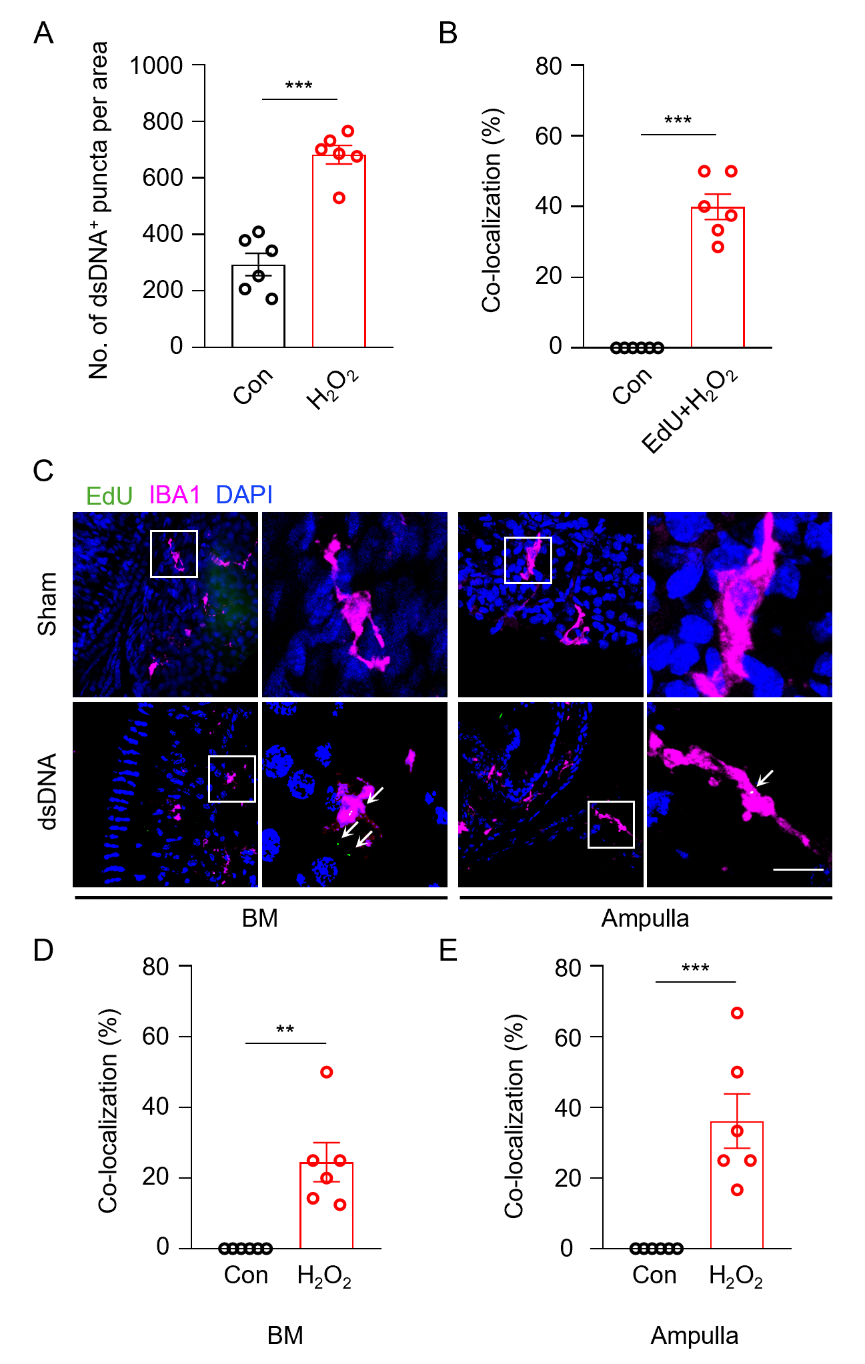


Fig. S2 The dsDNA secreted by oxidative stressed HEI-OC1 cells uptake by macrophages.

**(A)** Quantification of the cytosolic dsDNA puncta staining in H_2_O_2_ stimulated or control HEI-OC1 cells (n = 6 biologically independent experiments). ROIs were defined as 150 μm square. **(B)** Quantification of the percentage of VRMLs (CD68) that co-localizes with dsDNA (EdU) from PSCC-injected mice. ROIs were defined as 150 μm square. **(C)** Representative immunofluorescence images showing EdU (green), IBA1 (magenta) and DAPI (blue) staining in the BMs and ampullae of mice PSCC injected with EdU-labeled HEI-OC1 cells dsDNA and sham controls (n = 6 biologically independent experiments, scale bar = 12·5 μm). The right panels represent 4-fold enlargements of the white-boxed areas in the left panels. White arrows indicate dsDNA puncta. Quantification of the percentage of macrophages (IBA1) that co-localizes with dsDNA (EdU) from BMs **(D)** and ampullae **(E)** of PSCC injected mice. BM, basilar membrane. VRMLs, vestibular-resident macrophage-like cells. Results are presented as mean ± SEM. **P* < 0·05; ***P* < 0·01; ****P* < 0·001, by analysis of two-tailed unpaired Student's *t*-test.


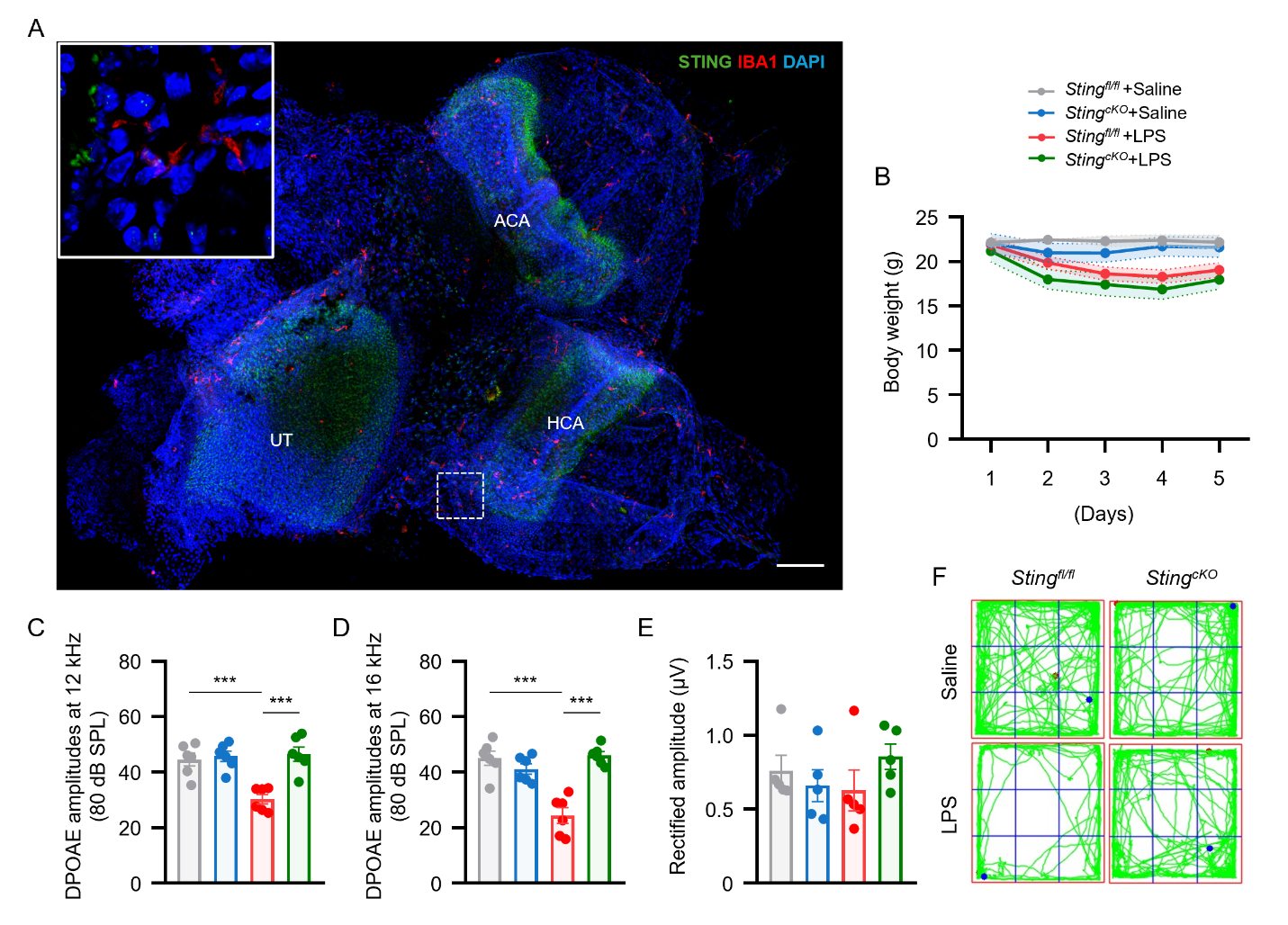


Fig. S3 LPS-induced EH and audio-vestibular symptoms were attenuated in *Sting* conditional knockout mice. (A) Representative immunofluorescence images showing STING (green), IBA1 (red) and DAPI (blue) staining in the ampullae and utricles of *Sting^cKO^* mice (n =5 mice per group, scale bar = 100 μm). (B) Changes of body weight in all groups in saline- or LPS-treated *Sting^fl/fl^* and *Sting^cKO^* mice (n =5 mice per group). DPOAE amplitudes at 12 kHz (C) and 16 kHz (D) of saline- or LPS-treated *Sting^fl/fl^* and *Sting^cKO^* mice (n =6 mice per group) (E) Changes in P1-N1 peak amplitudes at 100 dB nHL in saline- or LPS-treated *Sting^fl/fl^* and *Sting^cKO^* mice (n =5 mice per group). (F) Representative tracks of saline- or LPS-treated *Sting^fl/fl^* and *Sting^cKO^* mice in open-field tests during 10 min tracking period (n =5 mice per group). UT, utricle; ACA, anterior cristae ampulla; HCA, horizontal cristae ampulla. Results are presented as mean ± SEM. **P* < 0·05; ***P* < 0·01; ****P* < 0·001, by analysis of one-way ANOVA followed by Tukey's test.


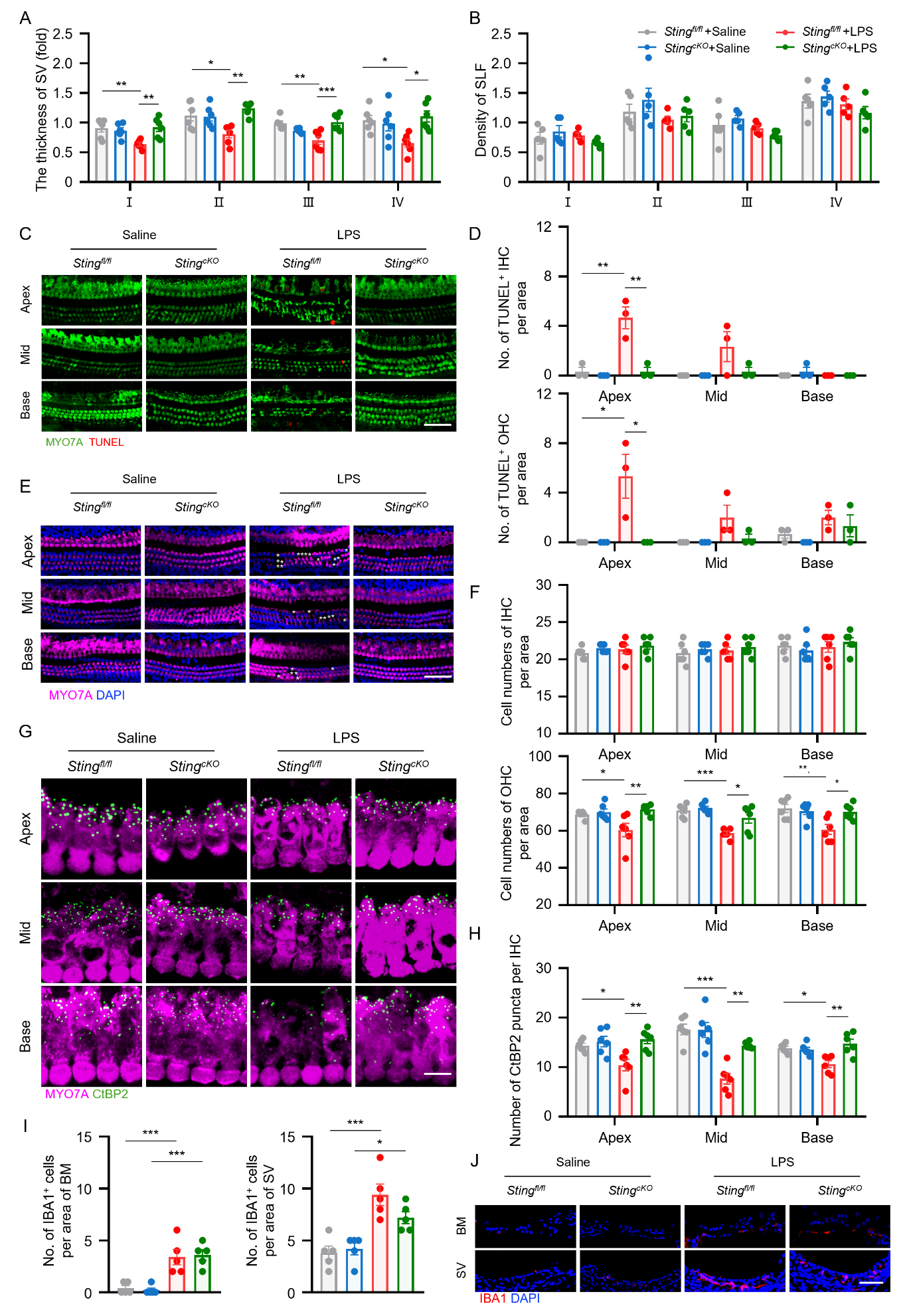


**Fig. S4 LPS-induced EH and audio-vestibular symptoms were attenuated in *Sting* conditional knockout mice. (A)** Quantification of the thickness of SV of saline or LPS-treated *Sting^fl/fl^* and *Sting^cKO^* mice (n =6 mice per group). **(B)** Quantification of the density of SLF per 100 μm^2^ of saline- or LPS-treated *Sting^fl/fl^* and *Sting^cKO^* mice (n =5 mice per group). (**C**) Representative immunofluorescence images showing MYO7A (green) and TUNEL (red) staining in the BMs of saline- or LPS-treated *Sting^fl/fl^* and *Sting^cKO^* mice (n  = 3 mice per group, scale bar = 50 μm) **(D)** Quantification of the number of TUNEL^+^ cells per area in the BM of saline- or LPS-treated *Sting^fl/fl^* and *Sting^cKO^* mice (n =3 mice per group, scale bar = 50 μm). ROIs were defined as 150 μm square. (**E**) Representative immunofluorescence images showing MYO7A (magenta) and DAPI (blue) in BMs of saline- or LPS-treated *Sting^fl/fl^* and *Sting^cKO^* mice. White asterisks indicate hair cell loss (n =3 mice per group, scale bar = 50 μm). (F) Quantification of the number of IHC and OHC per area. (G) Representative immunofluorescence images showing MYO7A (magenta) and CtBP2 (green) in IHC of saline- or LPS-treated *Sting^fl/fl^* and *Sting^cKO^* mice (n =6 mice per group, scale bar = 50 μm). (H) Quantification of the number of CtBP2 puncta per IHC. **(I)** Quantification of the number of IBA1^+^ macrophages per area. ROIs were manually drawn based on structures of BMs and SVs. **(J)** Representative confocal microscopy images showing IBA1 (red) and DAPI (blue) staining in BMs and SVs of saline- or LPS-treated *Sting^fl/fl^* and *Sting^cKO^* mice (n =5 mice per group, scale bar = 50 μm). BM, basilar membrane. SV, stria vascularis. SLF, spiral ligament type IV fibrocytes. Results are presented as mean ± SEM. **P* < 0·05; ***P* < 0·01; ****P* < 0·001, by analysis of one-way ANOVA followed by Tukey's test.


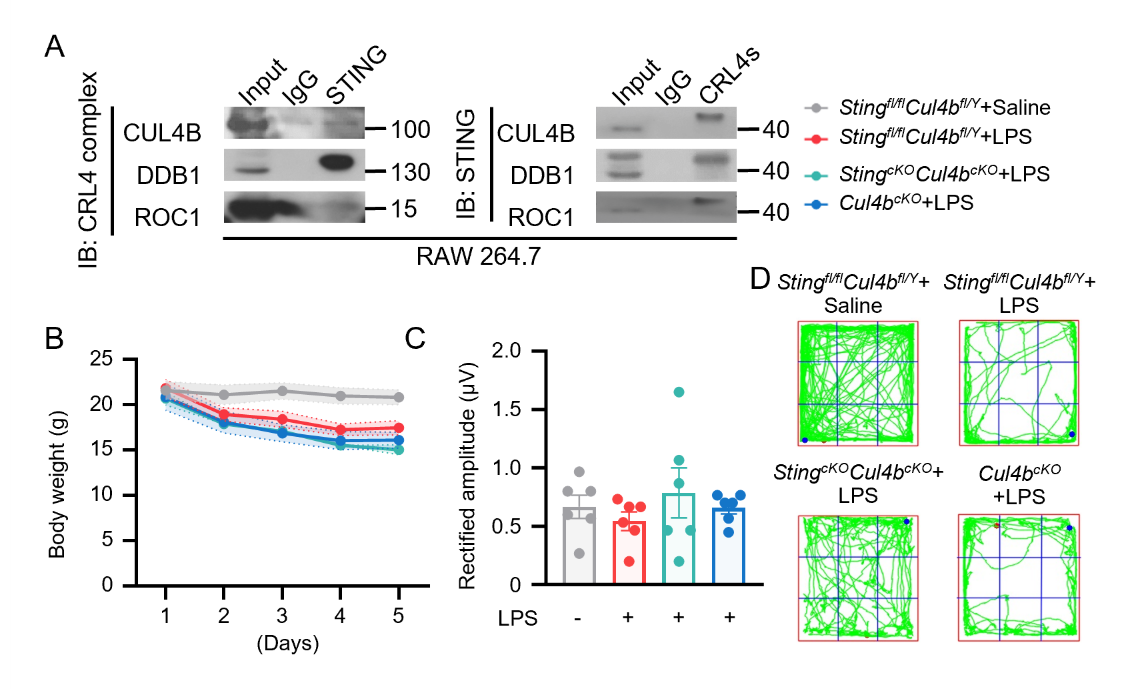


**Fig. S5 *Sting* knockout alleviates LPS-induced audio-vestibular symptoms, which are aggravated by *Cul4b* deficiency.** (**A**) Co-IP analysis of the interaction of STING and CRL4Bs in RAW264·7 cells. (**B**) Changes of body weight in all groups in saline- or LPS-treated *Sting^fl/fl^Cul4b^fl/Y^*, *Cul4b^cKO^* and *Sting^cKO^Cul4b^cKO^* mice (n =6 mice per group). (**C**) Changes in P1-N1 peak amplitudes at 100 dB nHL in saline- or LPS-treated *Sting^fl/fl^Cul4b^fl/Y^*, *Cul4b^cKO^* and *Sting^cKO^Cul4b^cKO^* mice (n =6 mice per group). (**D**) Representative tracks of saline- or LPS-treated *Sting^fl/fl^Cul4b^fl/Y^*, *Cul4b^cKO^* and *Sting^cKO^Cul4b^cKO^* mice in open-field tests during 10 min tracking period (n =5 mice per group). Results are presented as mean ± SEM. **P* < 0·05; ***P* < 0·01; ****P* < 0·001, by analysis of one-way ANOVA followed by Tukey's *t*est.

Table S1. Clinical features of patients with MD/VS.

| **Variables** | **MD (n=15)** | **VS (n=12)** | **P value** | **Effect size (95% CI)** |
| --- | --- | --- | --- | --- |
| Age, Mean (SD) | 64.0 (8.5) | 58.7 (7.5) | 0.101 | 0.10 (-1.11, 11.78) |
| Gender (%women) | 8 (53.3%) | 9 (75%) | 0.4244 | 2.62 (0.51, 11.44) |
| Age of onset, Mean (SD) | 52.6 (13.2) | 50.2 (6.8) | 0.5759 | 0.01 (-6.31, 11.05) |
| Time course (years), mean (SD) | 11.4 (18.2) | 8.5 (8.7) | 0.6122 | 0.01 (-8.86, 14.76) |
| Side, n (%) | | | | |
| Unilateral MD (UMD) | 14 (93.3%) | N/A | - | - |
| Bilateral MD (BMD) | 1 (6.7%) | N/A | - | - |
| Clinical subtype, n (%) | | | | |
| UMD 1, classical MD | 8 (53.3%) | N/A | - | - |
| UMD 2, delayed MD | 1 (6.7%) | N/A | - | - |
| UMD 4, migraine | 1 (6.7%) | N/A | - | - |
| UMD 5, autoimmune disease | 2 (12.3%) | N/A | - | - |
| BMD 1, metachronic sensorineural hearing loss | 2 (12.3%) | N/A | - | - |
| BMD 4, migraine | 1 (6.7%) | N/A | - | - |
| PTA at diagnosis, mean (SD) | 88.2 (25.1) | 73.3 (25.2) | 0.1402 | 0.08 (-5.22, 34.89) |
| Word recognition scores, mean (SD) | 16.5% (19.6) | 33.3% (33.4) | 0.1154 | 0.09 (-0.38, 0.04) |
| cVEMP abnormal, n (%) | 12 (80%) | 5 (41.7%) | 0.0568 | 0.17 (0.04, 0.96) |
| oVEMP abnormal, n (%) | 13 (86.7%) | 5 (41.7%) | 0.0369 | 0.10 (0.02, 0.72) |
| Tumarkin crisis, n (%) | 5 (33.3%) | 0 | 0.047 | 0 (0.00, 0.79) |
| High blood pressure, n (%) | 7 (46.7%) | 2 (16.7%) | 0.2172 | 0.22 (0.04, 1.38) |
| Type 2 diabetes, n (%) | 4 (26.7%) | 1 (8.3%) | 0.3419 | 0.25 (0.01, 2.13) |
| Autoimmune disease, n (%) | 2 (13.3%) | 1 (8.3%) | >0.999 | 0.59 (0.03, 5.71) |
| Migraine, n (%) | 2 (13.3%) | 1 (8.3%) | >0.999 | 0.59 (0.03, 5.71) |
| Functional level, n% | | | | |
| 1 | 0 | N/A | - | - |
| 2 | 1 (6.7%) | N/A | - | - |
| 3 | 0 | N/A | - | - |
| 4 | 2 (12.3%) | N/A | - | - |
| 5 | 7 (46.7%) | N/A | - | - |
| 6 | 5 (33.3%) | N/A | - | - |

MD, Ménière's Disease; VS, vestibular schwannoma; UMD, unilateral Ménière’s disease; BMD, bilateral Ménière’s disease; PTA, pure tone average of 0.5, 1, 2 and 4 kHz; WRS, word recognition scores; cVEMP, vestibular evoked myogenic potential; oVEMP, ocular vestibular evoked myogenic potential. CI, confidence interval. Grading of functional levels based on the Functional Level Scale (1-6) defined in the 1995 Committee on Hearing and Equilibrium Guidelines. Results are analysis of Fisher's exact test and two-tailed unpaired Student's *t*-test. Effect sizes were calculated as odds ratios (OR) with 95% CI for Fisher's exact test, and as eta squared (η²) with 95% CI for Student's *t*-test. N/A, not applicable.

Table S2. RT-PCR Primers

| GENE | PRIMER |
| --- | --- |

| human-*IFNB*-F | ATGACCAACAAGTGTCTCCTCC |
| --- | --- |
| human-*IFNB*-R | GGAATCCAAGCAAGTTGTAGCTC |
| human-*IL6*-F | ACTCACCTCTTCAGAACGAATTG |
| human-*IL6*-R | CCATCTTTGGAAGGTTCAGGTTG |
| human-*MX2*-F | CAGAGGCAGCGGAATCGTAA |
| human-*MX2-*R | TGAAGCTCTAGCTCGGTGTTC |
| human-*CXCL10*-F | GTGGCATTCAAGGAGTACCTC |
| human-*CXCL10*-R | TGATGGCCTTCGATTCTGGATT |
| human-*CCL5*-F | CCAGCAGTCGTCTTTGTCAC |
| human-*CCL5*-R | CTCTGGGTTGGCACACACTT |
| human-*IFIT1*-F | AAAGCAGGACCCACAAGAAT |
| human-*IFIT1*-R | CACCATTTGTACACATCTCCACT |
| human-*IFIT2*-F | TGAAAGAGCGAAGGTGTGCT |
| human-*IFIT2*-R | CTCAGAGGGTCAATGGCGTT |
| human-*IFIT3*-F | AAAAGCCCAACAACCCAGAAT |
| human-*IFIT3*-R | CGTATTGGTTATCAGGACTCAGC |
| human-*ISG15*-F | CGCAGATCACCCAGAAGATCG |
| human-*ISG15*-R | TTCGTCGCATTTGTCCACCA |
| human-*USP18*-F | AACGTGCCCTTGTTTGTCCAA |
| human-*USP18*-R | GAGTCCTTCACCCGGATCGTA |
| human-*MX1*-F | GGTGGTCCCCAGTAATGTGG |
| human-*MX1*-R | CGTCAAGATTCCGATGGTCCT |
| mouse-*Ifnb*-F | CAGCTCCAAGAAAGGACGAAC |
| mouse-*Ifnb*-R | GGCAGTGTAACTCTTCTGCAT |
| mouse-*Il6*-F | TAGTCCTTCCTACCCCAATTTCC |
| mouse-*Il6*-R | TTGGTCCTTAGCCACTCCTTC |
| *Continued* |  |
| GENE | PEIMER |
| mouse-*Cxcl10*-F | CCAAGTGCTGCCGTCATTTTC |
| mouse-*Cxcl10*-R | GGCTCGCAGGGATGATTTCAA |
| mouse-*Ccl5*-F | GCTGCTTTGCCTACCTCTCC |
| mouse-*Ccl5*-R | TCGAGTGACAAACACGACTGC |
| mouse-*Ifit1*-F | AAGGCTGTCCGGTTAAATCCA |
| mouse-*Ifit1*-R | TGAAGAGCTTTGTCTACGCGA |
| mouse-*Ifit2*-F | CTCTTTTATCCAGAACCCACCC |
| mouse-*Ifit2*-R | CAGCACAGAGTTGAGAGGTTGT |
| mouse-*Ifit3*-F | TCAGGCTTACGTTGACAAGGT |
| mouse-*Ifit3*-R | CACACTTTAGGCGTGTCCATC |
| mouse-*Isg15*-F | TCTTTCTGACGCAGACTGTAGA |
| mouse-*Isg15*-R | GGGGCTTTAGGCCATACTCC |
| mouse-*Usp18*-F | CAGGAGTCCCTGATTTGCGTG |
| mouse-*Usp18*-R | CAGAGGCTTTGCGTCCTTATC |
| mouse-*Mx1*-F | CCTCCCACATCTGTAAATCACTG |
| mouse-*Mx1*-R | CGGTTTCCTGTGCTTGTATCA |
| mouse-*Mx2*-F | GAGGCTCTTCAGAATGAGCAAA |
| mouse-*Mx2*-R | CTCTGCGGTCAGTCTCTCT |

Data S1. (separate file)

Mass spectrometry results of STING-combined protein in HEK-293T cells.

Data S2. (separate file)

LC-MS/MS results of the specific GlyGly (K)Sites of STING in HEK-293T cells.
